# Inhibitory activity of quercetin, its metabolite, and standard antiviral drugs towards enzymes essential for SARS-CoV-2: the role of acid–base equilibria†

**DOI:** 10.1039/d0ra09632f

**Published:** 2021-01-12

**Authors:** Žiko B. Milanović, Marko R. Antonijević, Ana D. Amić, Edina H. Avdović, Dušan S. Dimić, Dejan A. Milenković, Zoran S. Marković

**Affiliations:** University of Kragujevac, Faculty of Science, Department of Chemistry 12 Radoja Domanovića 34000 Kragujevac Serbia; University of Kragujevac, Institute for Information Technologies, Department of Science Jovana Civijića bb 34000 Kragujevac Serbia zmarkovic@uni.kg.ac.rs; Juraj Strossmayer University of Osijek, Department of Chemistry Ulica cara Hadrijana 8/A Osijek Croatia; University of Belgrade, Faculty of Physical Chemistry Studentski trg 12-16 11000 Belgrade Serbia

## Abstract

The recently declared global pandemic of a new human coronavirus called SARS-CoV-2, which causes respiratory tract disease COVID-19, has reached worldwide resonance and global efforts are being made to look for possible cures. Sophisticated molecular docking software, as well as available protein sequence and structure information, offer the ability to test the inhibition of two important targets of SARS-CoV-2, furin (FUR) enzyme, and spike glycoprotein, or spike protein (SP), that are key to host cell adhesion and hijacking. The potential inhibitory effect and mechanism of action of acid–base forms of different antiviral drugs, dominant at physiological pH, chloroquine (CQ), hydroxychloroquine (HCQ), and cinanserin (CIN), which have been shown to be effective in the treatment of SARS-CoV-2 virus, is reported with the special emphasis on their relative abundances. On the other hand, the potential inhibitory effect of the dominant acid–base forms of quercetin (Q) and its oxidative metabolite 2-(3,4-dihydroxybenzoyl)-2,4,6-trihydroxy-3(2*H*) benzofuranone (BZF), which are constituents of traditional food products believed to exhibit antiviral effects, was also examined. The undertaken study includes the determination of the major energy contributions to the binding energy as well as in-depth analysis of amino acid residues at the active pocket and possible interactions. The approach that we propose here may be an additional strategy for combating the deadly virus by preventing the first step of the virus replication cycle. Preliminary research has shown that the investigated compounds exert an inhibitory effect against the SARS-CoV-2 furin enzyme and spiked glycoprotein through different acid–base forms. These investigations may be helpful in creating potential therapeutic agents in the fight against the SARS-CoV-2 virus. On the other hand, the results we predicted in this computational study may be the basis for new experimental *in vitro* and *in vivo* studies.

## Abstract 2

Identification of potential inhibitory effect of acid–base species of quercetin and its metabolite as well as chloroquine, hydroxychloroquine, and cinanserin, at physiological pH, on proteins essential for SARS-CoV-2 virus survival.

## Graphical abstract

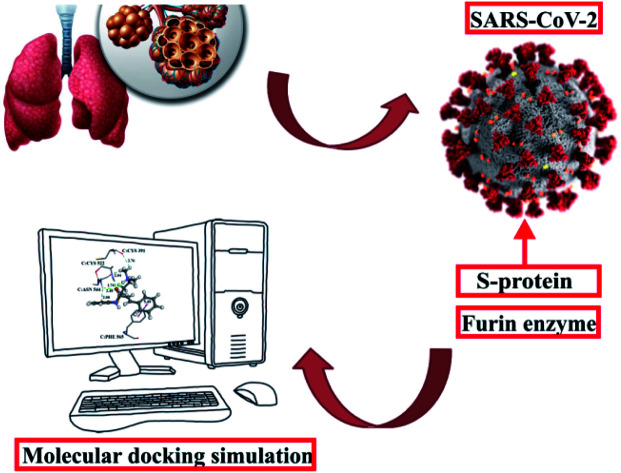

## 1. Introduction

In recent decades, several viral outbreaks have posed great threats to human health and society. In December 2019, human coronavirus disease (COVID-19) was reported in Wuhan, China, and was named SARS-CoV-2 by the International Committee for Virus Taxonomy.^1^ According to the World Health Organization (WHO), this disease was declared a global health emergency due to concerns over its increasing spread, and on March 11^th^ the disease was recognized as a pandemic.^2^ The SARS-CoV-2 virus is a member of *Betacoronaviruses* like the Severe Acute Respiratory Syndrome Human coronavirus (SARS HCoV) and the Middle-East Respiratory Syndrome Human coronavirus (MERS HCoV).^3,4^ A recent outbreak of novel coronavirus pneumonia caused by SARS-CoV-2 infection has spread rapidly around the globe. Although public health authorities are fighting the spread of COVID-19 viruses, the situation remains uncertain. As of November 11, 2020, there are more than 50 676 072 confirmed cases of COVID-19 and at least 1 261 075 reported deaths, indicating that it is a severe public health threat (https://covid19.who.int/).

The main problem with combating this infectious disease is that there are no vaccines available, and these will take many months to research and develop. For this reason, many scientists have resorted to examining the effect of various known and newly synthesized compounds as potential inhibitors of this virus, as evidenced by a large number of available scientific articles. Some of the strategies used in combating the previous coronaviruses might be considered as starting points in this quest, as suggested in the paper by Xiu and coworkers.^5^ The first line of treatment involves the inhibition of SARS-CoV-2 spike glycoprotein or spike protein (SP) by various potential antiviral drugs.^6,7^ These proteins help the coronaviruses to bind to the membrane of the human cells and infect through a receptor-mediated interaction. A recently identified host cell receptor, angiotensin-converting enzyme II (ACE2), is particularly important for viral entry.^5^ The SARS-CoV-2 virus has a specific structure that allows it to bind at about 10 times more tightly to host cell receptors, than the corresponding spike protein of the other coronaviruses.^8^ The SP contains a site that recognizes and becomes activated by an enzyme called furin (FUR).^9,10^FUR is a host-cell enzyme, responsible for the nonclathoin mediated fusion of membranes, which increases the probability of the entanglement of SP with ACE2.^5,11^FUR also belongs to the highly specific, calcium-dependent proprotein/prohormone convertases, represented in various human organs such as the liver, lungs, and small intestines.^12^ The fact that this enzyme resides in all of these human tissues means that the virus can potentially attack several organs at once.^13^ The specific inhibitors of furin could prevent the cleavage of spikes and syncytium, therefore suppressing the virus reproduction. The ongoing quest for the small molecule inhibitors of FUR and SP should result in the lowered probability of interaction between SP and ACE2 of the host cell.^14^

**Fig. 1:**
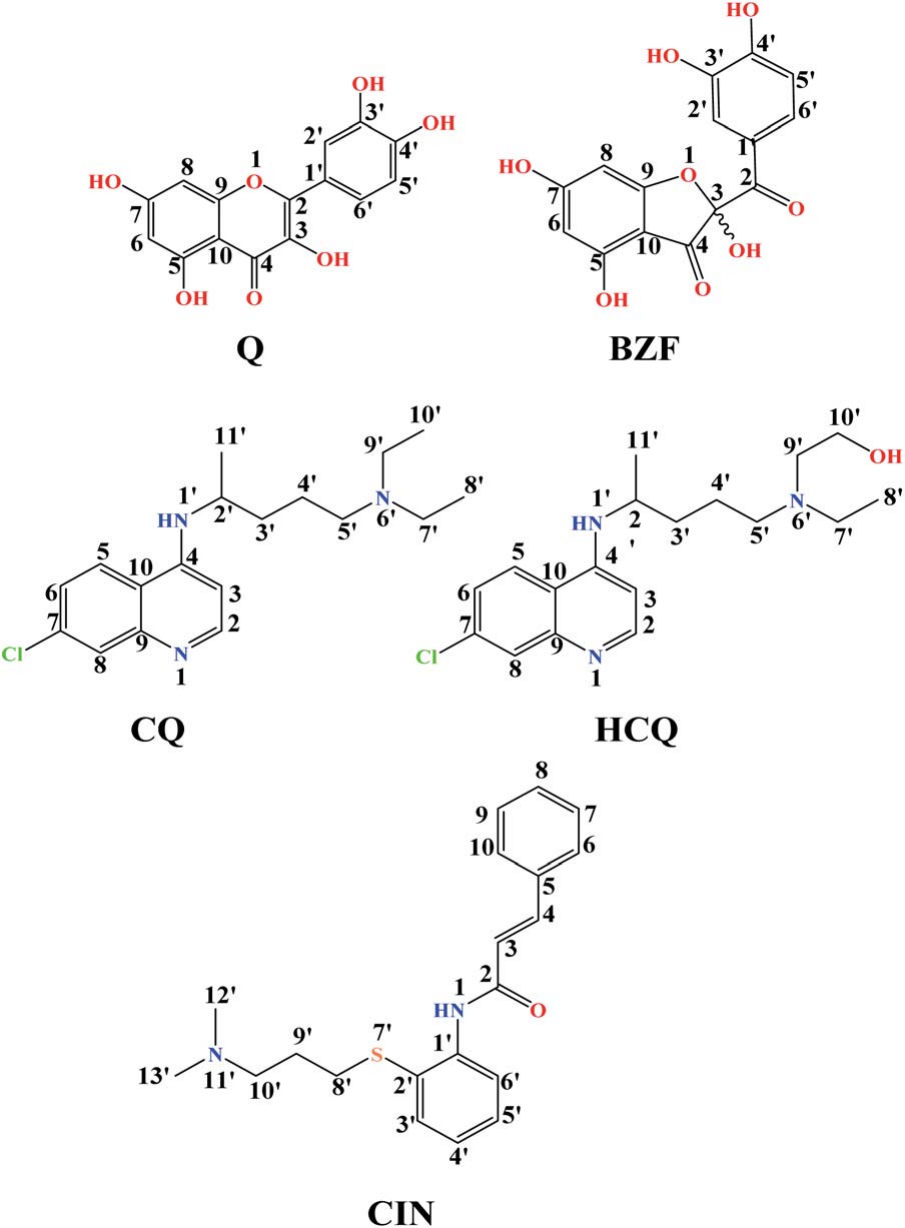
The structures of investigation compounds: quercetin (Q), 2-(3,4-dihydroxybenzoyl)-2,4,6-trihydroxy-3(2*H*)-benzofuranone(BZF), chloroquine (CQ), hydroxychloroquine (HCQ), cinanserin (CIN) with atomic numbering.

Quercetin (Q) and its major oxidation product its 2-(3,4-dihydroxybenzoyl)-2,4,6-trihydroxy-3(2*H*)-benzofuranone (BZF), is widespread in many plant species such as *Allium cepa*, *Oregano vulgare*, *Capsicum annum* which is believed to exhibit antiviral properties (Fig. 1).^15,16^ It has been shown to have a broad variety of biological activities and pharmacological actions such as virucidal effect against standbys virus, herpes simplex virus type 1 (HSV-l), and parainfluenza virus type 3 (Pf-3).^17^ Recent published results indicate that quercetin in combination with several different flavonoids could be involved in the treatment of multistage of the COVID-19 disease.^18^ The inhibitory effect of Q, along with three other tea polyphenols, was shown on biologically active human furin fragments by enzyme assays *in vitro*.^19^ On the other hand, several antimalarial drugs including chloroquine (CQ), hydroxychloroquine (HCQ), and cinanserin (CIN) (Fig. 1), were recently tested and showed apparent efficacy in treating SARS-CoV-2-associated pneumonia clinical studies.^20,21^ While much of the work was done on the experimental and theoretical analysis of the effect of these medications on the SARS-CoV-2 reproduction,^22^ the actual mechanism remains unclear. It is suggested that chloroquine and hydroxychloroquine, as weak bases, elevate the acidity of intracellular organelles, while cinanserin is an inhibitor of 3C-like proteinase of SARS-CoV-2.^23,24^ These compounds were selected along with Q and BZF because they bear some of the structural similarities, such as the fused rings and the presence of electronegative atoms which could be protonated/deprotonated depending on the pH of the medium. Some of the acid–base forms of the approved drugs could be responsible for the interactions with the chosen proteins.

It is known that the pH value of the medium affects the biological activity of different chemical compounds due to the presence of different acid–base forms of the investigated compounds. Assessment of the molar fraction (*f*) of the acid–base forms of the investigated compounds at physiological pH = 7.4 (blood serum) is vital for a proper understanding of their activity.^25^ This is especially important for drugs because ionic acid–base forms increase solubility in the aqueous phase while neutral forms are susceptible to passive transfer through biological membranes.^26^

In this study, the focus is on the examination of the potential inhibitory activity of Q and its metabolite BZF towards FUR and SP receptors necessary for SARS-CoV-2 virus survival and replication.

Also, the inhibitory activity of antimalarial drugs against the mentioned receptors was tested under the same conditions. Unlike other studies, our focus is on the estimation of the mole fraction and the investigation of the inhibitory effect of the dominant acid–base species of these compounds occurring at physiological pH.

The results of the inhibitory activity of Q and BZF with the selected antimalarial drugs will allow a deeper biochemical analysis for the possible application of combination therapy in the treatment of the SARS-CoV-2 virus. Discussion of the type and number of the binding interaction is analyzed to establish the structural requirements for the effective inhibition of FUR and SP enzymes.

## 2. Methodology

### 2.1. Determination of the molar fractions (*f*) of the acid–base forms of the investigated compounds

Molecules with more than one hydroxyl group, at physiological pH (pH = 7.4), can exist in more than one acid–base form. Since p*K*_a_ values can be treated as the measure of acidity, dissociation of investigated compounds depends on p*K*_a_ values. Based on the number of phenolic –OH groups and the structures of Q and BZF, five p*K*_a_ values can be expected. Therefore, the proportion of different forms of Q and BZF would vary depending on the pH of the environment. For Q, the deprotonation order, as well as the p*K*_a_ values, were experimentally determined by Álvarez-Diduk *et al.*^27^ Due to the lack of experimental data for BZF, p*K*_a_ values were determined by the ACD/p*K*_a_ software package, using the p*K*_a_ db program included in this software package.^28^

Antimalarial drugs that are effective in inhibiting the SARS-CoV-2 virus may exist in physiological pH in more than one acid–base form, due to the presence of nitrogen atoms in them, which are good proton acceptors. CQ, HCQ, and CIN are weak bases that can exist in protonated and unprotonated forms. The corresponding p*K*_a_ values for chloroquine (CQ, CQ^+^, CQ^2+^) were experimentally determined by Al-Bari.^29^ For hydroxychloroquine (HCQ, HCQ^+^, HCQ^2+^) and cinanserin (CIN, CIN^+^) the p*K*_a_ values were determined using the software mentioned above.

To estimate which of the above mentioned acid–base forms is dominant at physiological pH, it is necessary to calculate the molar fractions (*f*). Generally, for all polyprotic acids (H_*n*_A), the molar fraction of a fully deprotonated compound – anion fully deprotonated anion or a fully protonated compound-protonated cation is calculated by the formula:1
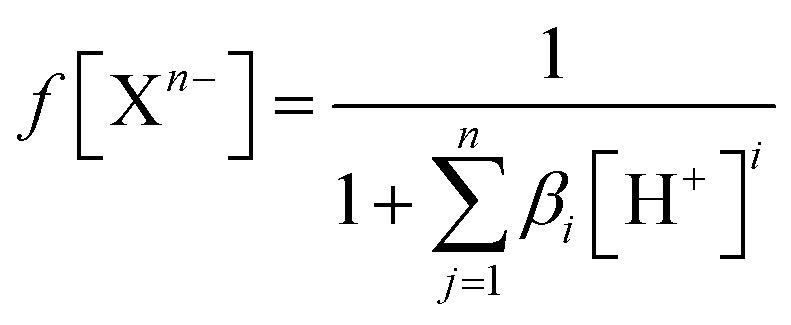
while the molar fraction of all other acid–base species is expressed by the following formula:

2 *f*[H_*i*_X^(*n*−1)−^] = *β*_*i*_[H^+^]^*i*^*f*(X^*n*−^)

where *β*_*i*_ is a global formation equilibrium constants:3
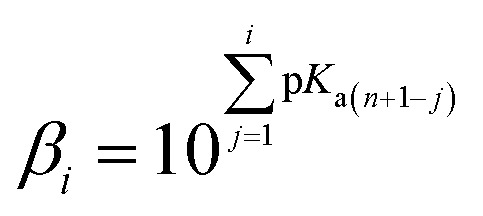


At physiological pH = 7.4, the concentration of H^+^ ion is 3.98 × 10^−8^ M.^30^

### 2.2. Molecular docking simulation

The equilibrium geometries of all investigated species were determined in water. DFT calculations were performed using the DFT/M06-2X/6-311++G(d,p) theoretical model in combination with the CPCM solvation model.^31,32^ All molecule and ion structures were optimized using the *Gaussian 09* software package.^33^

The AutoDock 4.0 software package with AMBER force field was employed to predict the scoring and binding interactions between the FUR and SP receptors and all dominant acid–base species of investigated compounds at physiological pH.^34^ The monomer crystal structure of FUR (PDB: 5JXG, 1.80 Å X-ray resolution) and quaternary structure of SP (PDB:6VSB, 2.71 Å electron microscopy) were extracted from the RCSB Protein Data Bank in PDB format (PDB).^35,36^ PDB file structures consist of heavy atoms and include co-crystallized ligands, water molecules, metal ions, and cofactor which could interfere with calculations. Based on a detailed analysis of results, it was found that the structures lacked connectivity information, which would have to be granted, along with bond orders and formal charges. To prepare and ensure the chemical correctness of protein structures in a form suitable for modeling calculations the Discovery Studio 4.0 (BIOVIA Discovery Studio 2016) was used.^37^ Also, this program was utilized for analysis and visualization of the obtained docking results after the simulation.

The hydrogen module in AutoDockTools (ADT) graphical interface was used to add polar hydrogen atoms in proteins. Partial atomic charges were assigned using the Kollman united atom partial charges. The protonation of amino acids is an important parameter in this study, as the binding of investigated compounds was monitored at physiological pH value. The DockPrep function of the DOCK program was used, as it generates reasonable protonation states at the mentioned pH value. The semi-flexible docking method was used, therefore FUR and SP were investigated as rigid input receptor molecules, while investigated compounds and derivatives were set to be flexible ligands. For ligands, the single bonds were set to be rotatable. Besides, the translation and rotation of the ligand in the grid were allowed. Grid maps were computed using AutoGrid considered cuboid docking grid coordinates of dimension *X*_min_/*X*_max_ = 42/60, *Y*_min_/*Y*_max_ = 42/60, *Z*_min_/*Z*_max_ = 40/60 with point separated by 0.375 Å (grid-point spacing). To generate orientations of ligands within the binding site of selected protein the Lamarckian Genetic Algorithm (LGA) were used with the following settings: the maximum number of energy evaluation was set to 2 500 000, a maximum of 1.0 × 10^6^ energy evaluations, the maximum number of generations 27 000, the maximum number of a top individual that automatically survived set to 1, a crossover rate of 0.80, a mutation rate of 0.02. A maximum of 10 conformers was considered for each investigated compound.

To predict the binding affinity, AutoDock uses empirical scoring functions based on the free energy of binding (Δ*G*_bind_).^38^ This equation includes different components. This value represents energy that is rid by the formation of interactions between a ligand and protein. The AutoDock program calculates this value according to the following equation:

4 Δ*G*_bind_ = Δ*G*_vdw+hbond+desolv_ + Δ*G*_elec_ + Δ*G*_total_ + Δ*G*_tor_ − Δ*G*_unb_

where Δ*G*_total_ is final total internal energy, Δ*G*_tor_ is torsional free energy, Δ*G*_unb_ is unbound system's energy, Δ*G*_elec_ is electrostatic energy and Δ*G*_vdw+hbond+desolv_ is the sum of dispersion and repulsion (Δ*G*_vdw_), hydrogen bond (Δ*G*_hbond_), and desolvation (Δ*G*_desolv_) energy. Also, the sum of Δ*G*_vdw+hbond+desolv_ and Δ*G*_elec_ represents free intermolecular energy, Δ*G*_inter_.^39^

Another important parameter is the constant of inhibition (*K*_i_). This value is calculated in AutoDock after estimation of free energy binding, using the following equation:

5 *K*_i_ = exp(Δ*G*_binding_/*RT*)

where *R* is the gas constant (*R* = 1.99 cal mol^−1^ K^−1^), *T* is the room temperature (298.15 K), *K*_i_ is the constant of inhibition.^40,41^

## 3. Results and discussion

### 3.1. Acid–base equilibria at physiological pH

**Fig. 2:**
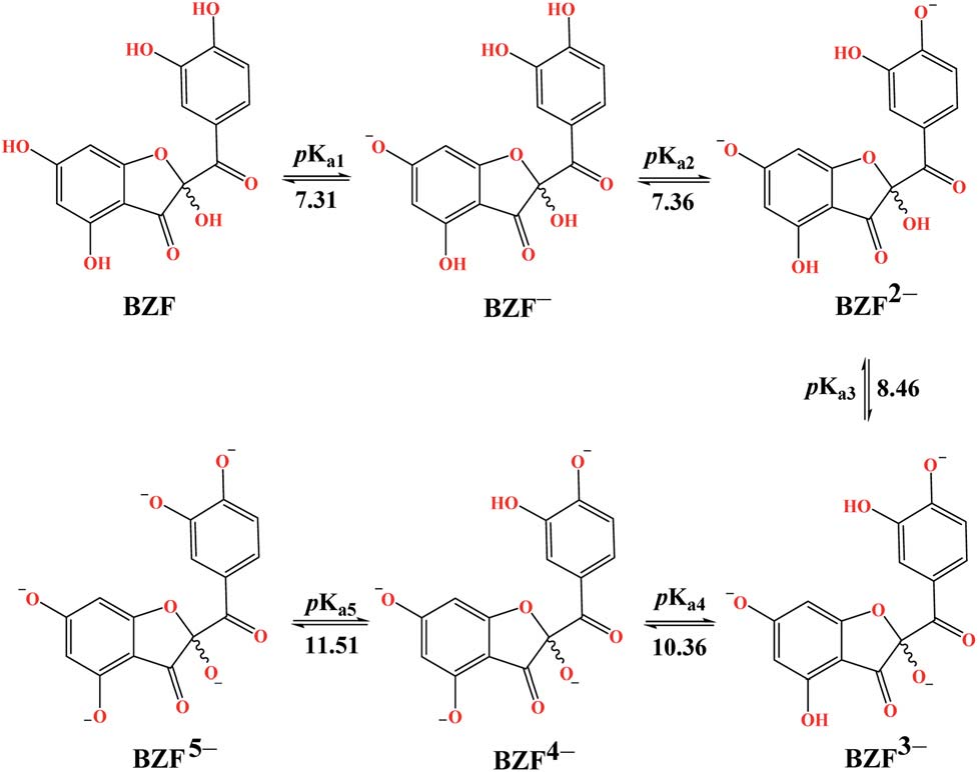
Predicted deprotonation process and corresponding p*K*_a_ values of BZF.

The experimentally determined order of Q deprotonation is 4′-OH, 7-OH, 3-OH, 3′-OH, and 5-OH (Fig. S1†). On the other hand, the theoretically determined order of deprotonation for BZF, as previously explained, is as follows: 7-OH, 4′-OH, 3-OH, 5-OH, 3′-OH (Fig. 2).

At physiological pH, the dominant species for Q and its metabolite BZF are monoanionic species (Table S1†), with a population of ∼66.72% and ∼36.24%, respectively. On the other hand, the significant proportions of neutral and dianionic acid–base forms of Q (6.67 and 66.72%, respectively) and BZF (29.54 and 31.56%, respectively) indicate that it is also necessary to examine the potential inhibitory effect of these species on the selected proteins as well.

**Fig. 3:**
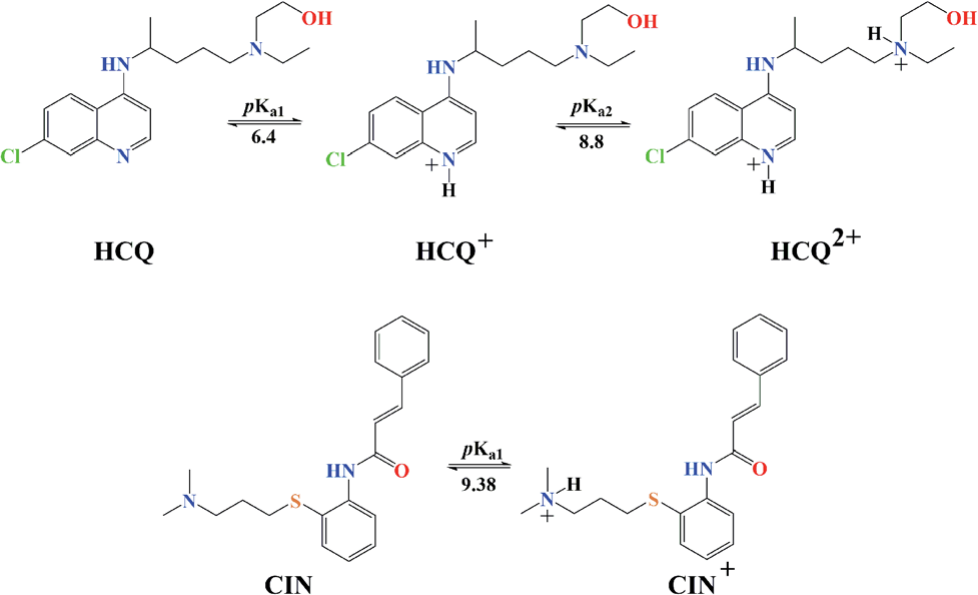
Predicted protonation process and corresponding p*K*_a_ values of HCQ and CIN.

The experimentally obtained p*K*_a_ values for CQ are presented in Fig. S2,† while the predicted protonation processes for HCQ and CIN are shown in Fig. 3. Also, the molar fractions of these antimalarial compounds are given in Table S1.† The presence of nitrogen atoms in structures of approved drugs allows the existence of protonated species. In the most significant proportion, CQ exists in the deprotonated form (83.24%) at physiological pH, while HCQ and CIN in monoprotonated (87.73 and 98.86%). All significantly proportioned acid–base forms were subjected to molecular docking simulations, and the results obtained were compared with those obtained for acid–base forms Q and BZF.

**Fig. 4:**
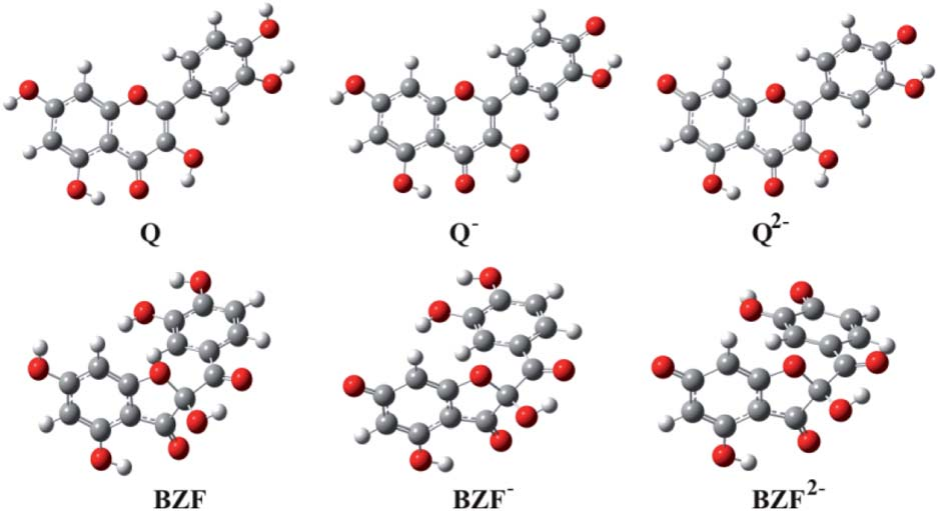
The optimized geometries of dominant acid–base forms of Q and BZF at M06-2X/6-311++G(d,p) level of theory (gray-carbon atoms, red-oxygen atoms, white-hydrogen atoms)

The optimized equilibrium structures of molecule, anion, and dianion of Q and BZF are presented in Fig. 4, while the geometries of the cations species of the standard drugs are presented in Fig. S3.† It is clear that the compounds in Fig. 4 contain several polar –OH groups as well as partially negatively charged oxygen (O^−^) atoms. The atoms of the mentioned functional groups represent significant donors or acceptor species in interactions with different amino acids of the selected proteins. On the other hand, the quinoline ring, the hydrocarbon sequence, and the present heteroatoms (N, Cl, S) allow a significant number of hydrophobic and electrostatic interactions of CQ, HCQ, and CIN with the amino acid residues of the proteins under study.

### 3.2. Molecular docking simulation-thermodynamic properties

The interactions between the acid–base species of Q and BZF with the FUR enzyme were first examined. The obtained thermodynamic parameters are given in Table 1 and Fig. S4.† The most stable conformations of the whole protein-ligand structures are presented in Fig. S5 and S6.† It is observed that the values of binding free energies (Δ*G*_bind_) and inhibition constants (*K*_i_) increase in the following order FUR–Q < FUR–Q^−^ < FUR–BZF^2−^ < FUR–BZF < FUR–BZF^−^ < FUR–Q^2−^, whereby the inhibitory effect decreases. The highest binding affinity was shown for the neutral form of Q (−7.77 kcal mol^−1^, 2.02 μM) and the dianionic form of BZF (−7.71 kcal mol^−1^, 2.24 μM). It is important to point out that Q and Q^−^ have similar values of thermodynamic parameters, while Q^2−^ has a value of binding energy that is lower for 1 kcal mol^−1^. With the formation of anionic species, the number of possible interactions in the active pocket probably decreases due to the presence of negatively charged groups. This is investigated in more detail in the following section. As shown previously all three acid–base forms of BZF are present in equal amounts and their binding energies are similar. Experimentally, the inhibition of biologically active human furin fragment (hfurin) by Q gave the *K*_i_ value of 23.27 μM.^42^ The difference in experimental and theoretical *K*_i_ values can be explained by the fact that only amino acids 108–573 are included in hfurin which led to the increased resistivity of the whole structure.^43^ The authors suggested that the auto-oxidation of quercetin and the formation of the reactive oxygen species could be the possible mechanism of the reaction. Also, this could mean that the oxidation products of Q are responsible for the binding to hfurin. BZF, the major oxidation product of Q,^44^ as shown by the two-electron electrochemical oxidation, in its most abundant state is equally potent as Q^−^ (Table 1), as obtained in this study.

**Table 1:** The important thermodynamic parameters for best docking conformations of investigated acid–base forms Q, BZF and standard drugs (CQ, HCQ and CIN) with FUR and SP

Conformations	Δ*G*_bind_	*K*_i_	Δ*G*_inter_	Δ*G*_vdw+hbond+desolv_	Δ*G*_elec_	Δ*G*_total_	Δ*G*_tor_	Δ*G*_unb_
FUR–Q	−7.77	2.02	−9.56	−9.35	−0.21	−2.75	1.79	−2.75
FUR–Q^−^	−7.72	2.21	−9.21	−8.89	−0.23	−2.35	1.49	−2.35
FUR–Q^2−^	−6.74	11.39	−7.94	−7.78	−0.15	−2.34	1.19	−2.34
FUR–BZF	−7.42	3.67	−9.50	−8.96	−0.54	−1.94	2.09	−1.94
FUR–BZF^−^	−7.40	3.79	−9.19	−7.77	−0.35	−2.35	1.79	−2.35
FUR–BZF^2−^	−7.71	2.24	−9.20	−8.84	−0.36	−1.39	1.49	−1.39
SP–Q	−5.15	167.9	−6.94	−6.33	−0.61	−3.68	1.79	−3.68
SP–Q^−^	−6.16	30.4	−7.65	−5.75	−1.90	−3.45	1.49	−3.45
SP–Q^2−^	−7.68	2.33	−8.88	−6.19	−2.69	−3.77	1.19	−3.77
SP–BZF	−6.72	11.84	−8.81	−8.01	−0.80	−2.47	2.09	−2.47
SP–BZF^−^	−7.89	1.65	−9.68	−9.54	−0.13	−2.01	1.79	−2.01
SP–BZF^2−^	−8.40	0.69	−9.89	−8.77	−1.13	−2.94	1.49	−2.94
FUR–CQ	−6.54	16.09	−8.93	−7.97	−0.96	−1.43	2.39	−1.43
FUR–CQ^+^	−6.94	8.13	−9.03	−7.87	−1.16	−0.80	2.09	−0.80
FUR–CQ^2+^	−7.41	3.73	−9.49	−8.22	−1.27	−0.55	2.09	−0.55
FUR–HCQ	−6.76	11.00	−9.75	−8.14	−1.61	−0.93	2.98	−0.93
FUR–HCQ^+^	−7.17	5.52	−9.86	−8.96	−0.90	−0.79	2.68	−0.79
FUR–HCQ^2+^	−6.53	16.29	−9.22	−7.99	−1.23	−1.89	2.68	−1.89
FUR–CIN	−7.83	1.83	−10.22	−10.22	0.00	−1.75	2.39	−1.75
FUR–CIN^+^	−8.04	1.29	−10.42	−9.21	−1.21	−1.01	2.39	−1.01
SP–CQ	−8.31	0.81	−11.69	−11.64	−0.05	−0.96	2.39	−0.96
SP–CQ^+^	−8.85	0.33	−11.24	−11.22	−0.01	−0.8	2.39	−0.8
SP–CQ^2+^	−8.71	0.41	−11.10	−11.10	0.01	−0.74	2.39	−0.74
SP–HCQ	−8.10	1.15	−11.09	−11.04	−0.05	−0.93	2.98	−0.93
SP-HCQ^+^	−8.11	1.15	−11.09	−11.05	−0.04	−0.79	2.98	−0.79
SP-HCQ^2+^	−8.90	0.30	−11.88	−11.8	−0.08	−0.9	2.98	−0.9
SP-CIN	−9.93	0.05	−12.31	−12.26	−0.06	−1.5	2.39	−1.5
SP-CIN^+^	−9.69	0.08	−12.07	−12.01	−0.06	−1.59	2.39	−1.59

**Fig. 5:**
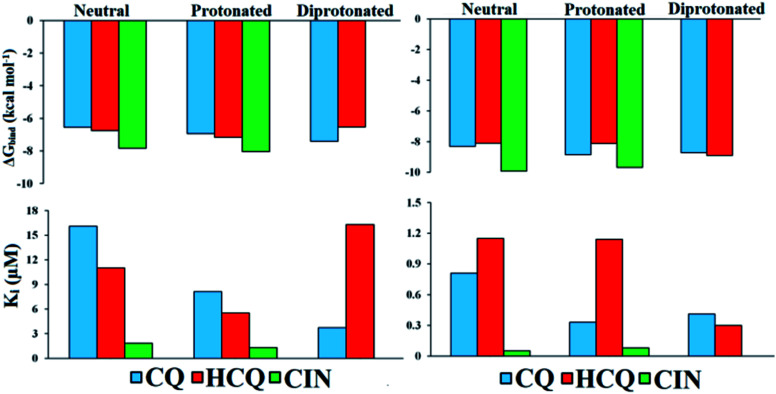
Histogram of important thermodynamic parameters after molecular docking simulation and estimation of CQ, HCQ, and CIN inhibitory activity according to FUR (left) and SP (right) enzymes.

Values of thermodynamic parameters for docking of standard drugs CIN, CQ, and HCQ with FUR enzyme are given in Table 1 and Fig. 5. The highest binding affinity was observed for the diprotonated form of chloroquine, CQ^2+^ (−7.41 kcal mol^−1^, 3.73 μM), and protonated forms of hydroxychloroquine, HCQ^+^ (−7.17 kcal mol^−1^, 5.52 μM), and cinanserin, CIN^+^ (−8.04 kcal mol^−1^, 1.29 μM). The protonated and most abundant acid–base form of CIN proved to be a better inhibitor of FUR enzymes than Q, BZF, and the other two drugs. On the other hand that acid–base forms Q and BZF exhibit competitive or better inhibitory activity in comparison to CQ and HCQ drugs.

The affinities of the acid–base species Q and BZF towards SP of the SARS-CoV-2 virus decrease in the following order SP–BZF^2−^ < SP–BZF^−^ < SP–Q^2−^ < SP–BZF < SP–Q^−^ < SP–Q. In both cases, the dianion forms show the highest inhibitory activity towards SP. The inhibitory activity of acid–base pairs of Q is reversed to that towards FUR, probably because more positive amino acid residues are present in SP. The standard drugs show a more pronounced inhibitory effect than acid–base forms of Q and BZF. The values for binding energies are between −8.10 and −9.93 kcal mol^−1^ which is significantly negative than for the two molecules discussed previously. The only BZF^2−^ with Δ*G*_bind_ of −8.40 kcal mol^−1^ is comparable to values calculated for approved drugs. The neutral and ionic forms of CIN show the highest binding activity out of three drugs. The correlation between the charge and binding constants is very complex in this case as it doesn't’ change uniformly for all investigated molecules. Therefore the various energy contributions are analyzed in the next paragraphs.

By careful analysis of the data given in Table 1, it can be concluded that the greatest contribution to binding free energy comes from the sum of dispersion and repulsion (Δ*G*_vdw_), hydrogen bond (Δ*G*_hbond_), and desolvation energy (Δ*G*_desolv_). In the case of FUR, the values of Δ*G*_vdw+hbond+desolv_ have spanned from −7.77 (FUR–BZF^−^) to −10.42 kcal mol^−1^ (FUR–CIN^+^). These values are comparable between Q and BZF on one side and CQ and HCQ on the other, while CIN has significantly higher values. A negligible contribution to the binding free energy comes from Δ*G*_elec_ electrostatic energy, with values in case of binding to FUR that are between 0 and −1.61 kcal mol^−1^. The values of Δ*G*_elec_ are much higher in the case of approved drugs than for Q and BZF, which could be the possible answer for the stronger binding. The nonspecific interactions, including hydrogen bonding, are almost the only contribution in the case of acid–base forms of these two compounds.

In the case of the SP protein, the situation is somewhat different. The Δ*G*_vdw+hbond+desol_ contributions in the case of Q and BZF are lower for several kcal mol^−1^. The proportion of electrostatic interactions is higher with actual values being between −0.6 and −2.69 kcal mol^−1^. The approved drugs have the values of binding energies for non-specific interactions with the amino acids in the active pocked which are higher than −11 kcal mol^−1^. These interactions are the only contribution, as the electrostatic ones contribute negligibly. The analysis of specific amino acids should give a better insight into the energy contributions of various interactions, but these results offer the possibility of the combined therapy that would target both of these proteins with compounds that show a higher affinity to a specific protein.

### 3.3. Molecular docking simulation-analysis of interactions

A detailed analysis of the interactions between the investigated acid–base forms and proteins essential for the survival of the SARS-CoV-2 virus allows a comprehensive interpretation of the mechanism of inhibitory action. The binding mode of protein–ligand complexes will be considered for those complexes that exhibit the best inhibitory effect.

**Fig. 6:**
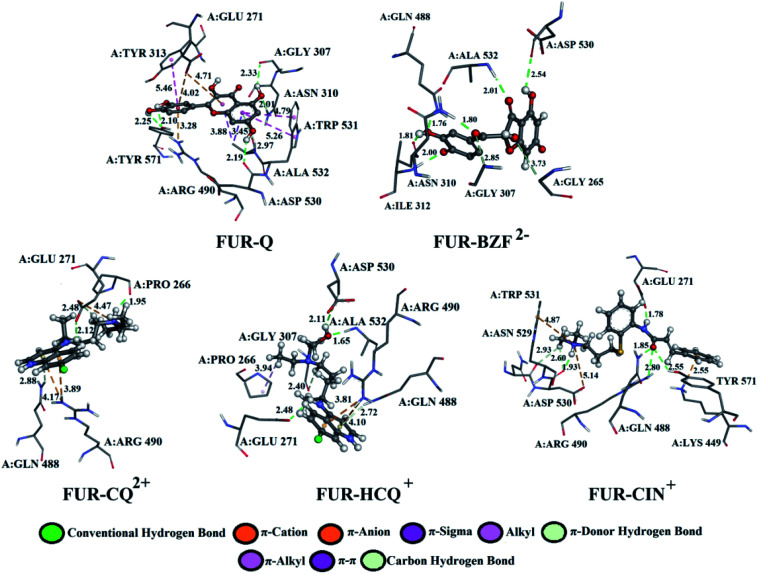
The best docking positions of different acid–base forms of investigation compounds to FUR enzyme.

At the active site of the FUR enzyme, the acid–base forms of Q and BZF (Fig. 6, S9, and Table S2†) are surrounded by similar amino acid residues as CQ, HCQ, and CIN (Fig. 6, S10 and Table S3†). This leads to the conclusion that the two groups of the investigated acid–base forms have a similar inhibitory mechanism on the FUR enzyme. All of the investigated compounds contain several polar groups, partially negative oxygen atoms (O^−^) of the carbonyl group and polar –OH groups (Q and BZF), as well as partially positive amino groups of neutral and cationic forms of the standard antimalarial drugs. Therefore, these compounds establish very significant nonspecific interactions-hydrogen bonds with the amino acid of residues such as A:GLY 307, A:ASN 310, A:LYS 449, A:ALA 532, A:TYR 571 in different positions. Hydrogen bond furcation is a ubiquitous phenomenon in macromolecular structures. A donor can interact with several acceptors simultaneously or an acceptor can interact simultaneously with many donors. The terms bifurcated and trifurcated are commonly used to describe these arrangements.^41,45^ The hydrogen atoms of the mentioned functional groups represent donor atoms that interact with different amino acids, while the partially charged heteroatoms N and O represent hydrogen atom acceptors.

There are two types of hydrogen bonds in the presented structures. The first type is conventional hydrogen bonds, with a bond length less than 3 Å, which have the most significant energy contribution to the Δ*G*_bind_ energy. The neutral form of quercetin acts both as donor and acceptor of hydrogen bonds. The oxygen atoms are acceptors of hydrogen bonds in interactions with A:ASN 310 (2.01 Å), while hydrogen atoms are donated to A:TYR 571 (bifurcated geometry, 2.25 and 2.10 Å), A:GLY 307 (2.33 Å), and A:ASP 530 (2.19 Å). Once Q^−^ is formed some of the hydrogen bonds are changed, and a new one is formed with A:LYS 449 (2.61 Å), and one of the bonds with A:TYR 571 is lost. Further deprotonation of this molecule, leads to the decrease in hydrogen bond number, namely only three are formed between Q^2−^ and A:ALA 532 (2.09 Å), A:TYR 313 (1.80 Å), and A:GLY 307 (3.05 Å). When values for the number of hydrogen bonds and their distances are correlated to the binding energies for acid–base from Table 1, it is clear that with the decrease in number and increase in distance the value of Δ*G*_bind_ decreases. BZF is a much more flexible molecule than Q with an additional oxygen atom, therefore the number of formed hydrogen bonds increases. A total of six conventional hydrogen bonds are formed with A:GLY 265, A:ASN 310, A:ALA 532, A:PRO 266, A:GLY 307, and A:GLY 271, all of which are below 3 Å. Only two amino acid residues are the same for neutral forms of Q and BZF. During the deprotonation of BZF, the total number of hydrogen bonds remains almost unchanged due to the flexibility of the molecule. The average bond length changes with deprotonation, 2.24 Å (BZF), 2.29 Å (BZF^−^), and 1.98 Å (BZF^2−^) which explains the relative order of the binding energies from Table 1.

The lower binding affinity of CQ then for Q and BZF and its acid–base forms can therefore be explained by the number of hydrogen bonds, as only one is present in complex FUR–CQ, none in FUR–CQ^+^, and three in FUR–CQ^2+^. The number of hydrogen bonds is between five and three for HCQ which led to similar binding energies to those of quercetin and its metabolite. The highest binding affinity towards FUR was calculated for CIN^+^ and in this case, a new type of hydrogen bond emerges in which the amino group acts as a donor with very short bond lengths, below 1.80 Å.

The second type of hydrogen bond is those formed with carbon atoms, carbon–hydrogen bond. These bonds have a length longer than conventional hydrogen bonds, which makes them weaker. In complex between FUR and Q^−^ there is one carbon–hydrogen, in complex with BZF^−^ one and with BZF^2−^ two, all of which are formed with glycine residues in positions 307 and 265 and with distance longer than 2.8 Å. The protonated forms of chloroquine form two carbon–hydrogen bonds with A:PRO 266 (2.51 and 2.55 Å) and A:GLU 271 (2.48 Å). HCQ and CIN establish one or two bonds of this type with different amino acids.

The most numerous interactions are hydrophobic or π-cation, π-anion, π–σ, π–π stacking, and π–π-T shaped. These interactions are characterized by the bond length of >3.0 Å. There are also attractive charges and salt bridges between CQ^+^ and A:GLU 271 (4.47 Å), HCQ^+^ and A:ASP 530 (2.99 Å), and CIN^+^ and A:ASP 530 (5.14 Å). Although hydrophobic interactions are numerous, from the analysis of the specific interactions, it is clear that the number and type of hydrogen bonds are determining factors for the stability of the formed complexes with FUR.

The results from Table 1 predict that the binding affinities of Q and BZF are lower than for the approved drugs.

**Fig. 7:**
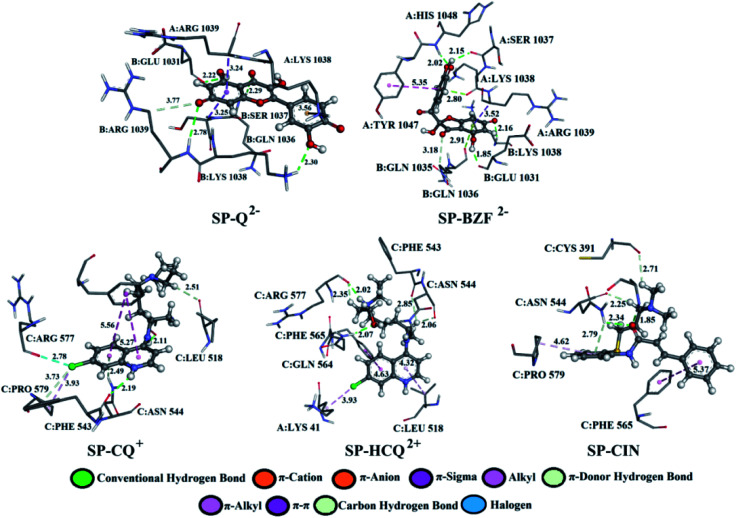
The best docking positions of different acid–base forms of investigated compounds to SP.

Q from three conventional hydrogen bonds (Fig. 7, S8, and Table S2†) with C:GLN 1036 (bifurcated geometry, 2.93 and 2.25 Å) and B:GLU 1031 (1.94 Å). When deprotonated, the number of hydrogen bonds increases to three (Q^−^) and four (Q^2−^). Also, an additional carbon–hydrogen bond is formed between Q^2−^ and B:ARG 1039 (3.77 Å). This explains the increase in binding affinity from −5.15 to −7.68 kcal mol^−1^ with deprotonation. A higher number of hydrogen and carbon–hydrogen bonds is observed in complexes between SP and BZF leading to higher stability of these complexes when compared to Q. Amino acid residues C:GLN 564 in SP–HCQ^2+^ (2.07 Å) and C:ASN 544 in SP–CIN (2.34 Å) complexes, establish conventional hydrogen bonds. Other types of hydrogen bonds are more dominant in the interactions of acid–base forms of standard drugs and SP receptors. Amino acid C:ASN 544, establishes a carbon–hydrogen bond with the acid–base forms HCQ^2+^ (2.06 Å) and CIN (2.25 Å). Also, hydrogen of the of –NH_2_ group of C:ASN 544 participates in the formation of the π-donor hydrogen bond with the aromatic quinoline ring CQ^+^ (2.49 Å) and CIN (2.79 Å). All of these interactions explain the affinity of approved drugs towards SP.

Interesting halogen interaction occurs between the σ-hole (positive electrostatic potential) of the chlorine atom of CQ^2+^ and the oxygen atom of amino acid C:ARG 577 (2.74 Å). The hydrogen atoms of the partially positive –NH– group of CQ^+^ and HCQ^2+^ establish a strong hydrogen bond with the oxygen atoms of amino acids C:PHE 543 (2.19 Å) and C:ARG 577 (2.02 Å). Another type of interaction is observed in the complexes of SP receptor and acid–base forms of BZF. Namely, the partially negative carbonyl group of amino acids B:GLN 1036 (2.91 Å) and A:LYS 1038 (2.80 Å) are in a specific position for the π-lone interactions with the aromatic ring BZF^2−^. The significance of this electrostatic interaction is reflected in the relatively high contribution of Δ*G*_elec_ (−1.13 kcal mol^−1^) in the Δ*G*_bind_ energy.

In all complexes, especially with the standards drugs, the most common type of interactions are hydrophobic interactions. The aromatic pyrone ring of compounds Q^2−^ (3.24 Å) and BZF^2−^ (3.52 Å) with electrons in σ-orbital A:ARG 1039 establishes a weak π*–*σ interaction. Also, low-range π–π-T-shaped contacts are established between the aromatic quinoline ring CQ^+^ (5.27 and 5.56 Å) and CIN (5.37 Å) and the phenyl ring of the amino acid C:PHE 565.

The analysis of specific amino acid residues and their interactions with investigated molecules proved the assumption that quercetin and its metabolite, as naturally occurring compounds could be used along with approved drugs as the binding affinities towards FUR are comparable, while approved drugs are still necessary for more specific proteins like SP.

The inhibition of furin by specific inhibitors occurs at the catalytically active position characterized by the strong hydrogen bond between triade: ASP153, HIS194, and SER368.^46^ The results from this study show that the investigated molecules are able to form the same type of interactions as the specific inhibitors, but further studies are needed on the actual mechanism through molecular dynamics studies. This would be additional proof that the binding of investigated molecules at the described sites induces changes in the native conformation of furin and lead to the distortion of the active site. It is also possible that the introduction of molecules in their different acid–base forms could inhibit the activation of spike protein and its binding to ACE2 due to the presence of stronger interactions between charged particles. Therefore, the authors strongly suggest the inclusion of different pH-dependent forms of analyzed inhibitors in the quest for the new COVID-19 medication.

## 4. Conclusions

The molecular docking study was used to evaluate the inhibitory activity of Q and its metabolite BZF, as well as standard antiviral drugs CQ, HCQ, and CIN on FUR and SP proteins essential for the replication and life cycle of the SARS-CoV-2 virus. Based on the experimental and calculated p*K*_a_ values, the most abundant acid–base forms. The obtained values of molar fractions indicate that Q, BZF, CQ, and HCQ express their inhibitory activity *via* three acid–base forms, while CIN*via* two. Thermodynamic parameters of protein binding prove the importance of the number and type of hydrogen bonds formed. With deprotonation of Q, the inhibitory activity towards FUR decreases (from −7.77 to −6.74 kcal mol^−1^) as the number of hydrogen bonds decreases and their lengths increase. In the case of BZF binding to FUR, the number of conventional hydrogen bonds is lower than for Q, which results in a lower affinity for all three acid–base forms. The affinity of CQ and HCQ is comparable to the two naturally occurring molecules, especially for the most active forms CQ^2+^ (−7.41 kcal mol^−1^) and HCQ^+^ (−7.17 kcal mol^−1^). The highest binding affinity towards FUR was calculated for CIN^+^ which is also the most abundant form at physiological pH values. In the case of approved drugs, important interaction is with the protonated amino acid characterized by the bond length of less than 1.8 Å. The main contribution to the binding energies comes from nonspecific interactions. On the other hand, the reactivity of Q and BZF towards SP is lower than for the investigated drugs which were proved by the lower number of hydrogen and carbon–hydrogen bonds. Again, the protonated form of CIN was the most effective compound. The results of this study indicate the potential therapeutic effect of the selected compounds through inhibition of FUR and SP. In summary, based on our comprehensive study, quercetin and its derivative, as naturally occurring compounds with a calculated affinity similar to an approved drug, may be the subject of future research as a potential combination therapy in the treatment of emerging COVID-19 disease.

## Conflicts of interest

The authors declare no competing financial interest.

## Supplementary Material

RA-011-D0RA09632F-s001

RA-011-D0RA09632F-s002
