# Supplementary material for: Inhibitory activity of quercetin, its metabolite, and standard antiviral drugs towards enzymes essential for SARS-CoV-2: the role of acid–base equilibria
Source: RSC Adv. 2021 Jan 12;11(5):2838–47. doi: 10.1039/d0ra09632f (PMC8693803; doi:10.1039/d0ra09632f)
Supplement: RA-011-D0RA09632F-s001 [file RA-011-D0RA09632F-s001.pdf]

Electronic Supplementary Material (ESI) for RSC Advances.
This journal is © The Royal Society of Chemistry 2021

Supplementary Material
for

Inhibitory activity of quercetin, its metabolite, and standard antiviral drugs towards enyzmes essential for SARS-CoV-2: the role of acid-base equilibria

Žiko Milanović[a], Marko Antonijević[b], Ana Amić[c], Edina Avdović[b], Dušan Dimić[d], Dejan Milenković[b], Zoran Marković[b*]

[a]*University of Kragujevac, Faculty of Science, Department of Chemistry, Radoja Domanovića 12, 34000 Kragujevac, Serbia*

[b] *University of Kragujevac, Institute for Information Technologies, Department of Science, 34000 Kragujevac, Serbia*

[c]*Juraj Strossmayer University of Osijek, Department of Chemistry, Ulica cara Hadrijana 8/A, Osijek, Croatia*

[d]*University of Belgrade, Faculty of Physical Chemistry, Studentski trg 12-16, 11000 Belgrade, Serbia*

[*]Corresponding author. Tel: +381 34 370270; Fax: +381 34 370168

E-mail address: zmarkovic@uni.kg.ac.rs (Zoran Marković)

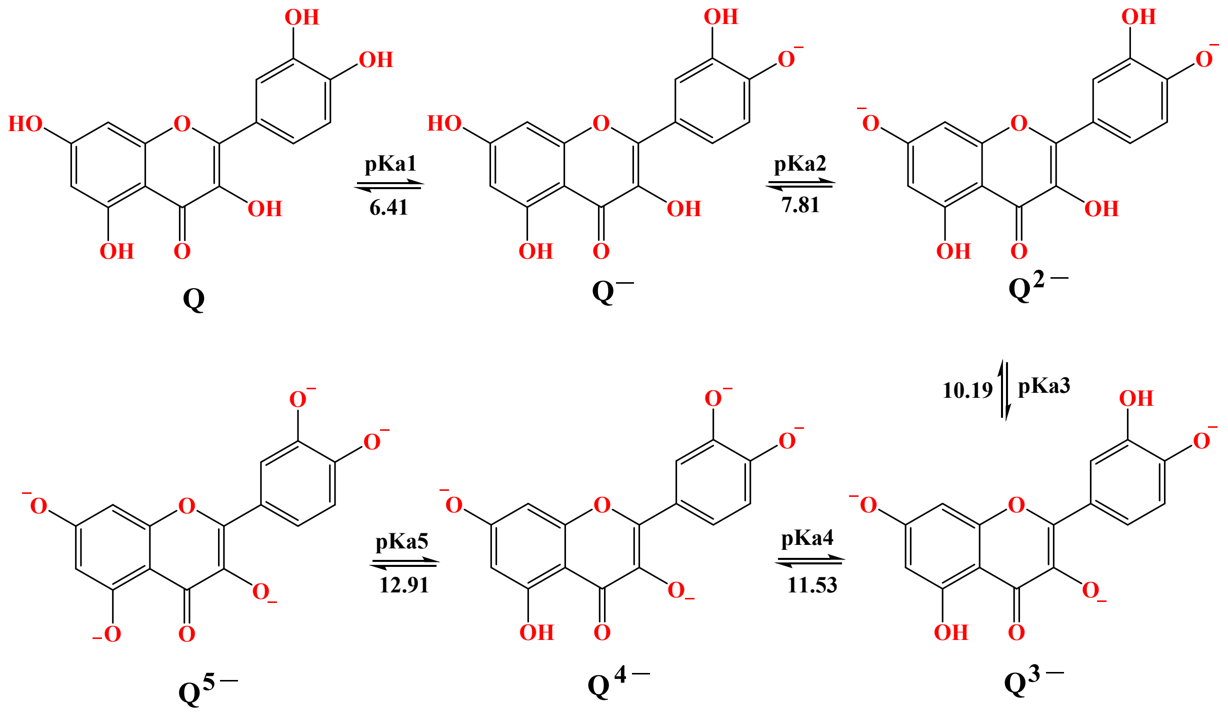

Fig. S1 Experimentally determined deprotonation process and corresponding $p\mathrm{K_a}$ values of **Q**

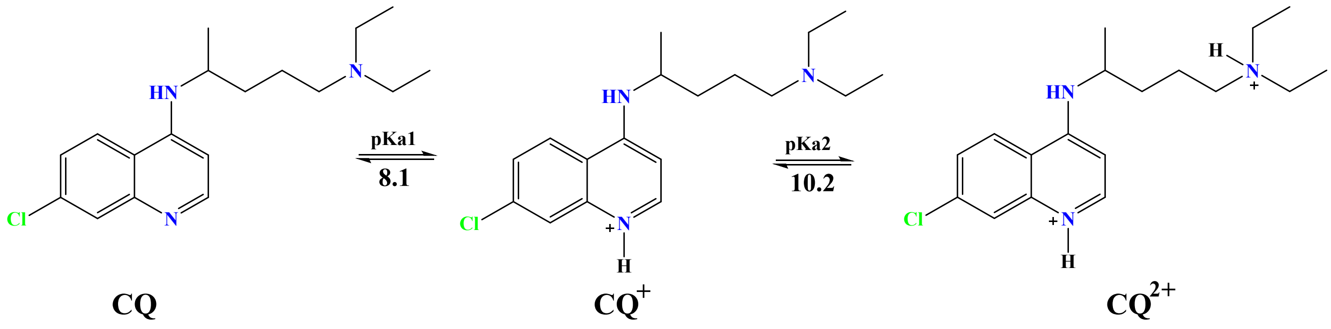

Fig. S2 Experimentally determined the protonation process and corresponding $p\mathrm{K_a}$ values of **CQ**

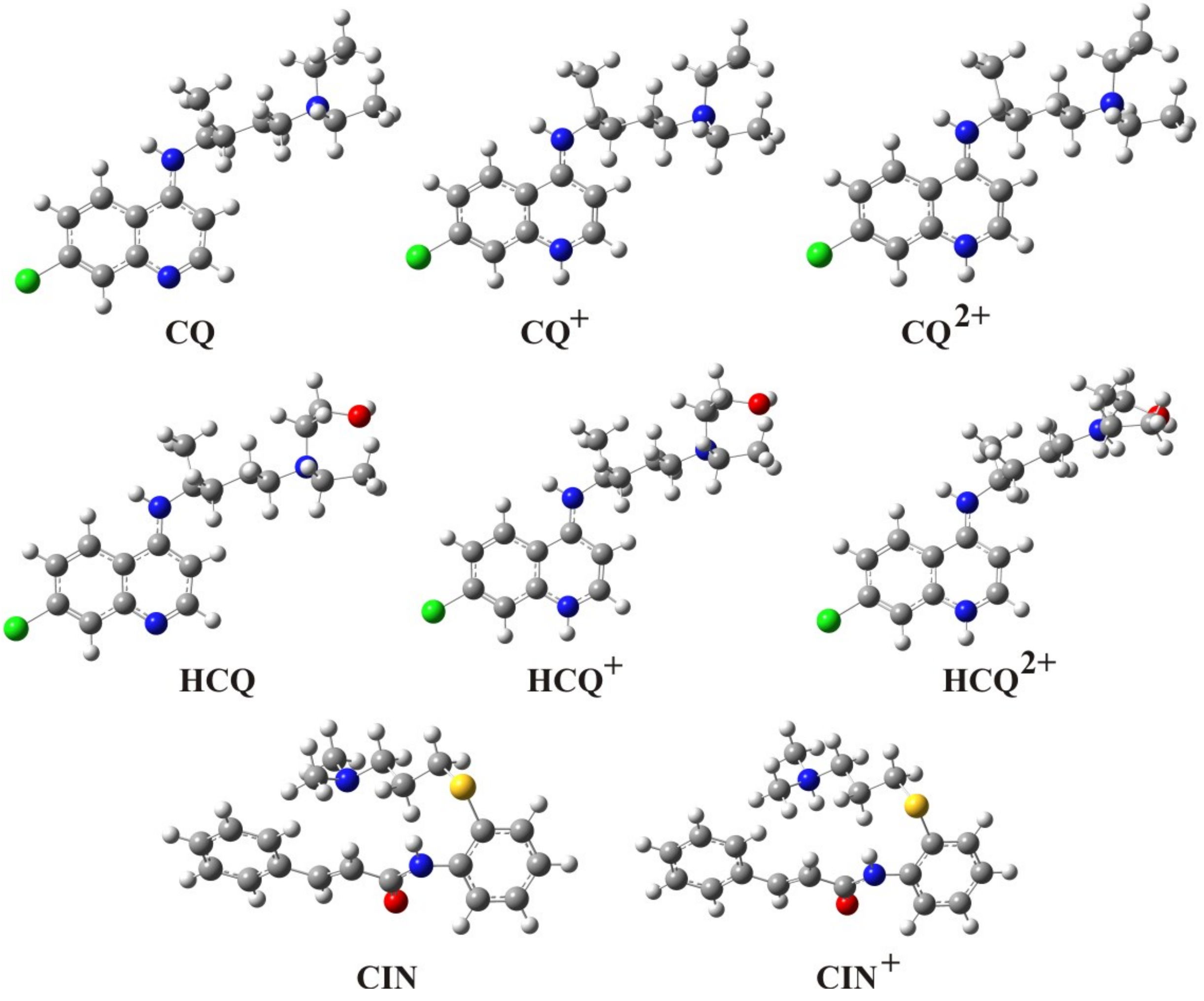

Fig. S3 The optimized geometries of dominant acid-base forms of **CQ**, **HCQ,** and **CIN** at M06-2X/6-311++G(d,p) level of theory

Table S1. Predicted molar fractions (*f*) of different acid-base species of investigated compounds at physiological pH

Investigation compounds*:	**R**	**R^-**	**R^{2-}**	**R^{3-}**	**R^{4-}**	**R^{5-}**
**Q**	6.67	66.72	26.56	0.04	0.00	0.00
**BZF**	29.54	36.24	31.56	2.75	0.00	0.00
Standards:	**R**		**R^+**		**R^{2+}**	
**CQ**	0.03		16.74		83.24	
**HCQ**	3.49		87.73		8.77	
**CIN**	1.04		98.86		/	

*R=investigation compounds

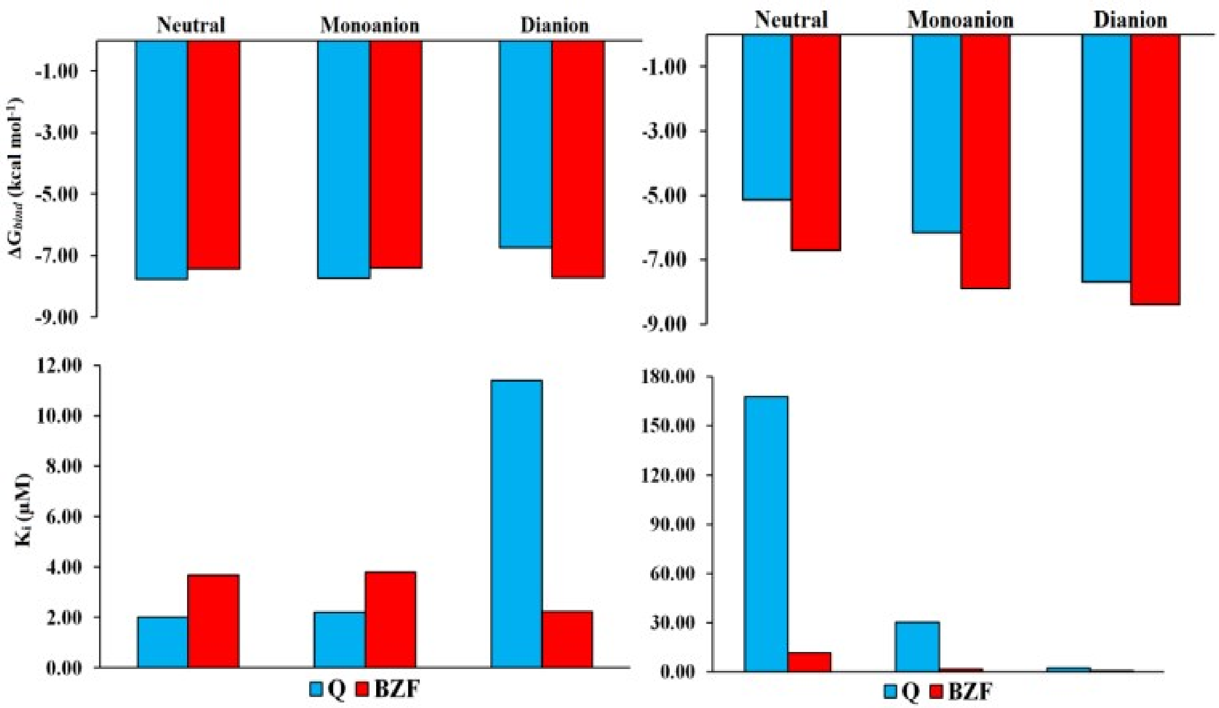

Fig. S4 Histogram of the important thermodynamic parameters for best docking conformations of investigated acid-base forms **Q** and **BZF** with **FUR** (left) and **SP** (right).

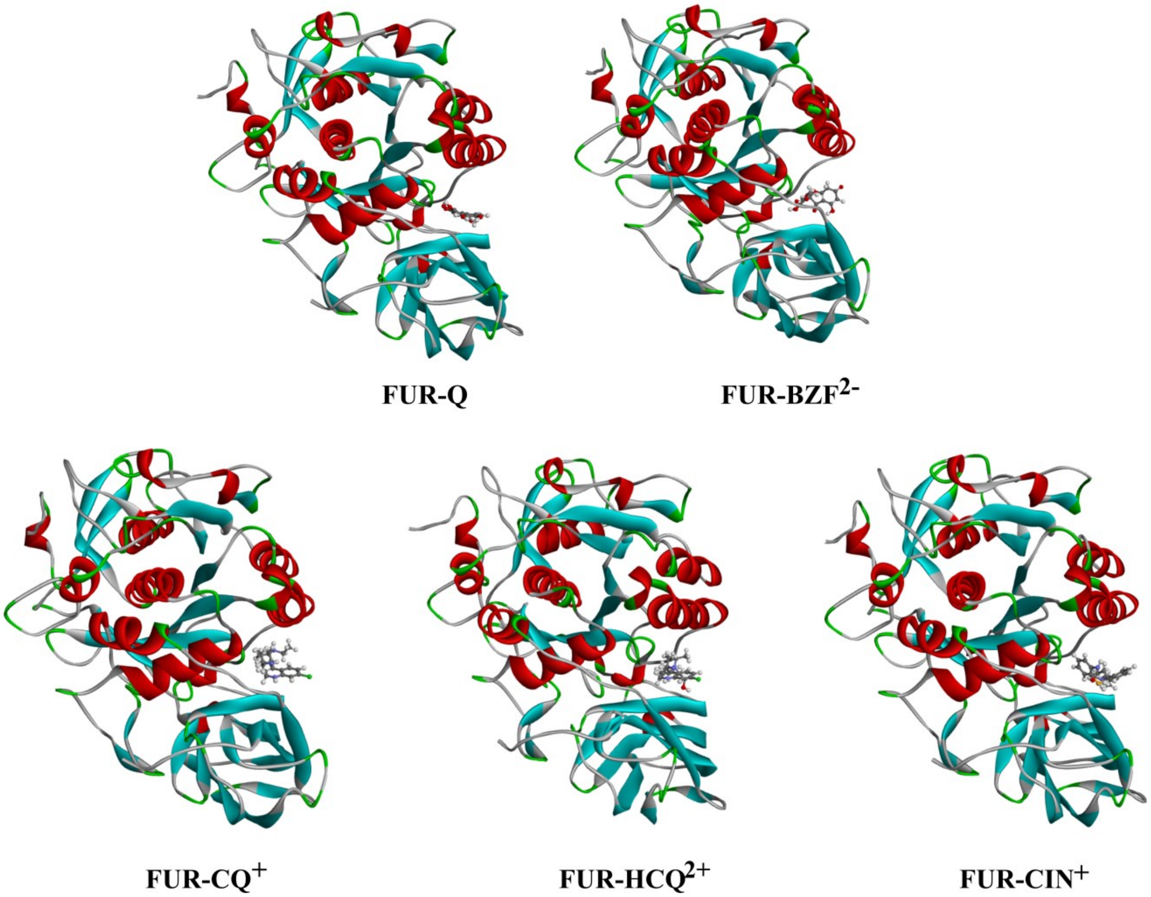

Fig. S5. 3D representation of the most stable **FU**R-ligand complexes obtained after a molecular docking study

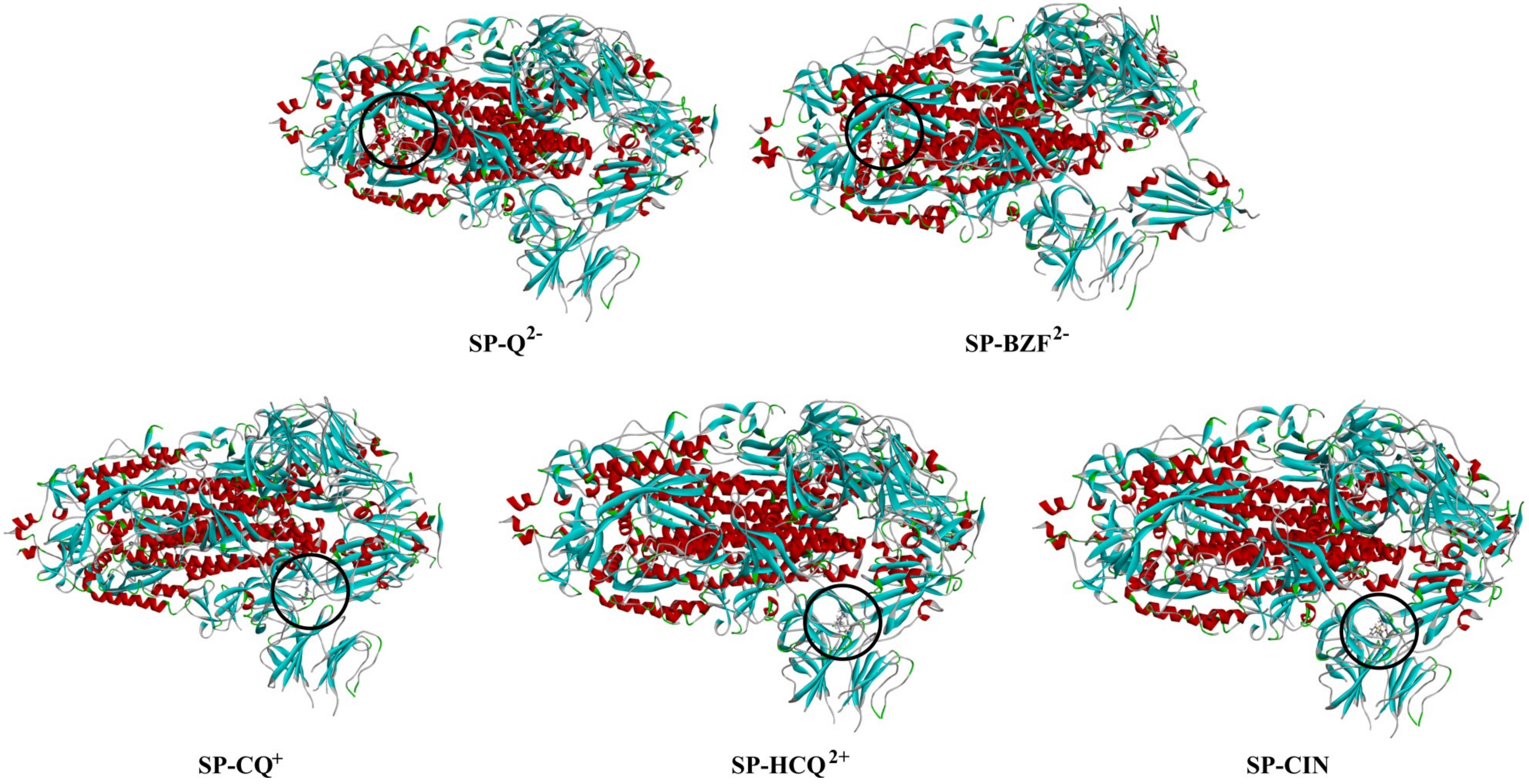

Fig. S6. 3D representation of the most stable **SP**-ligand complexes obtained after a molecular docking study (black circle-position of ligand)

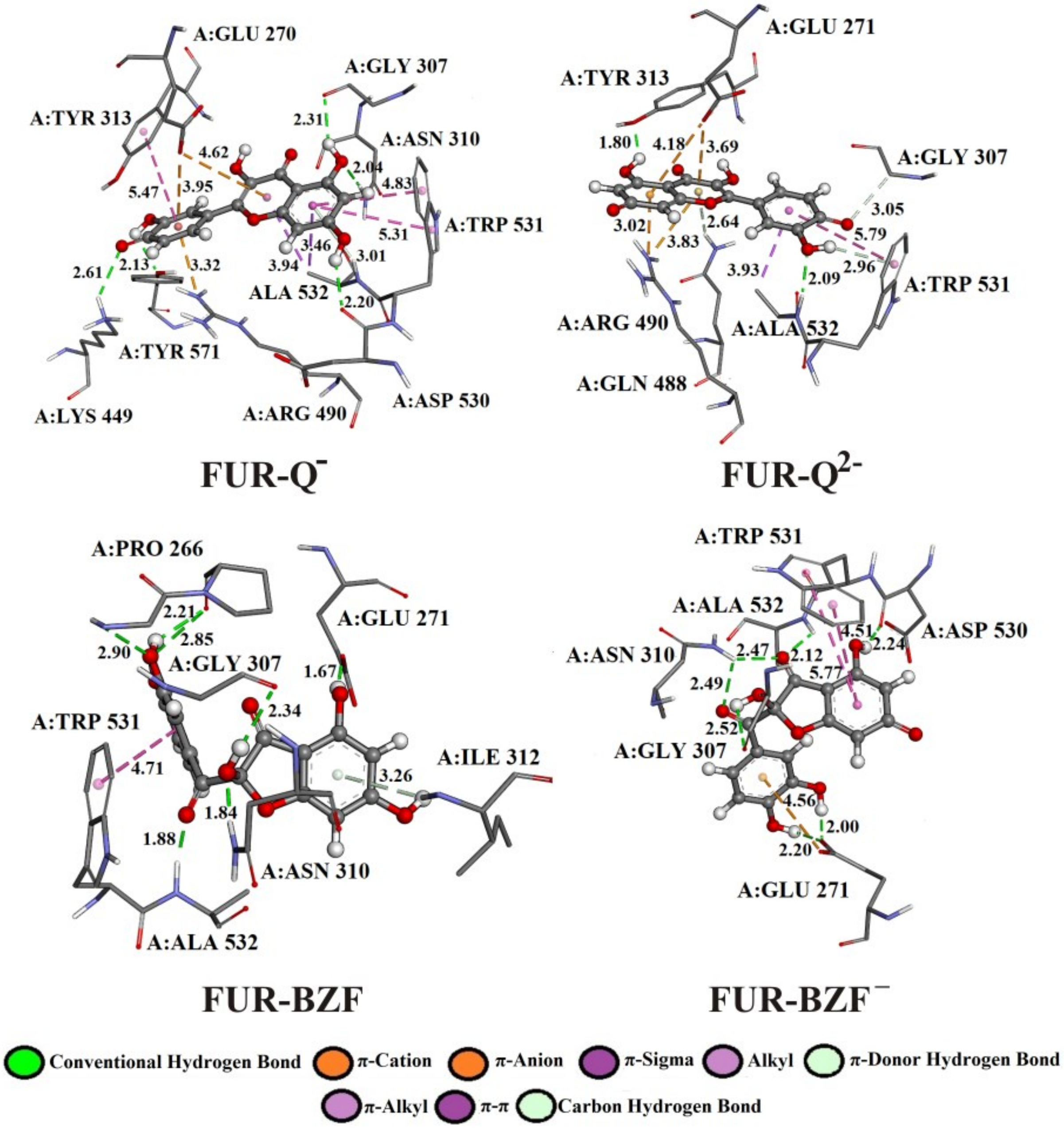

Fig. S7 The best docking positions of different acid-base forms of **Q** and **BZF** to **FUR** enzyme

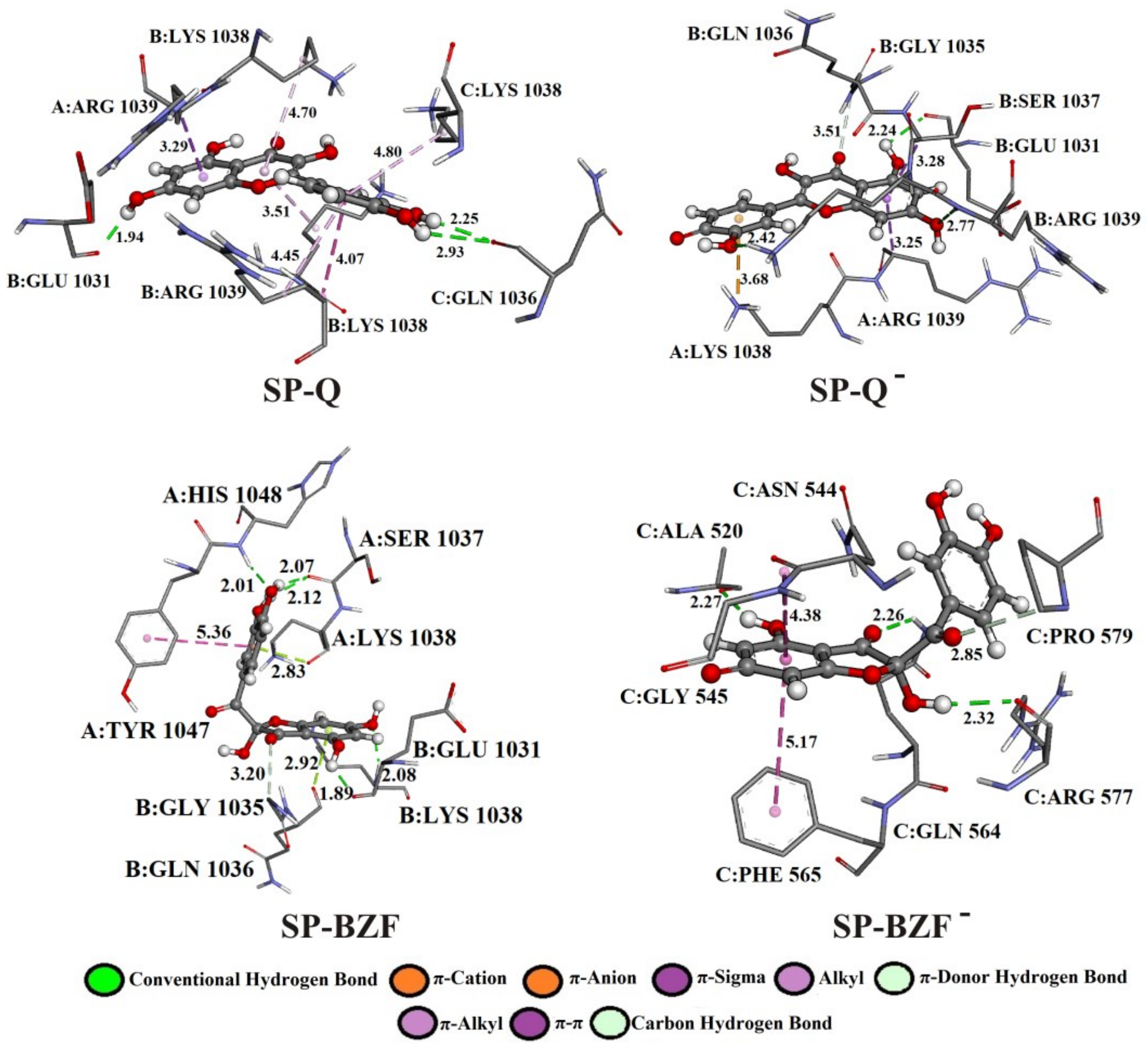

Fig. S8 The best docking positions of different acid-base forms of **Q** and **BZF** to **SP**

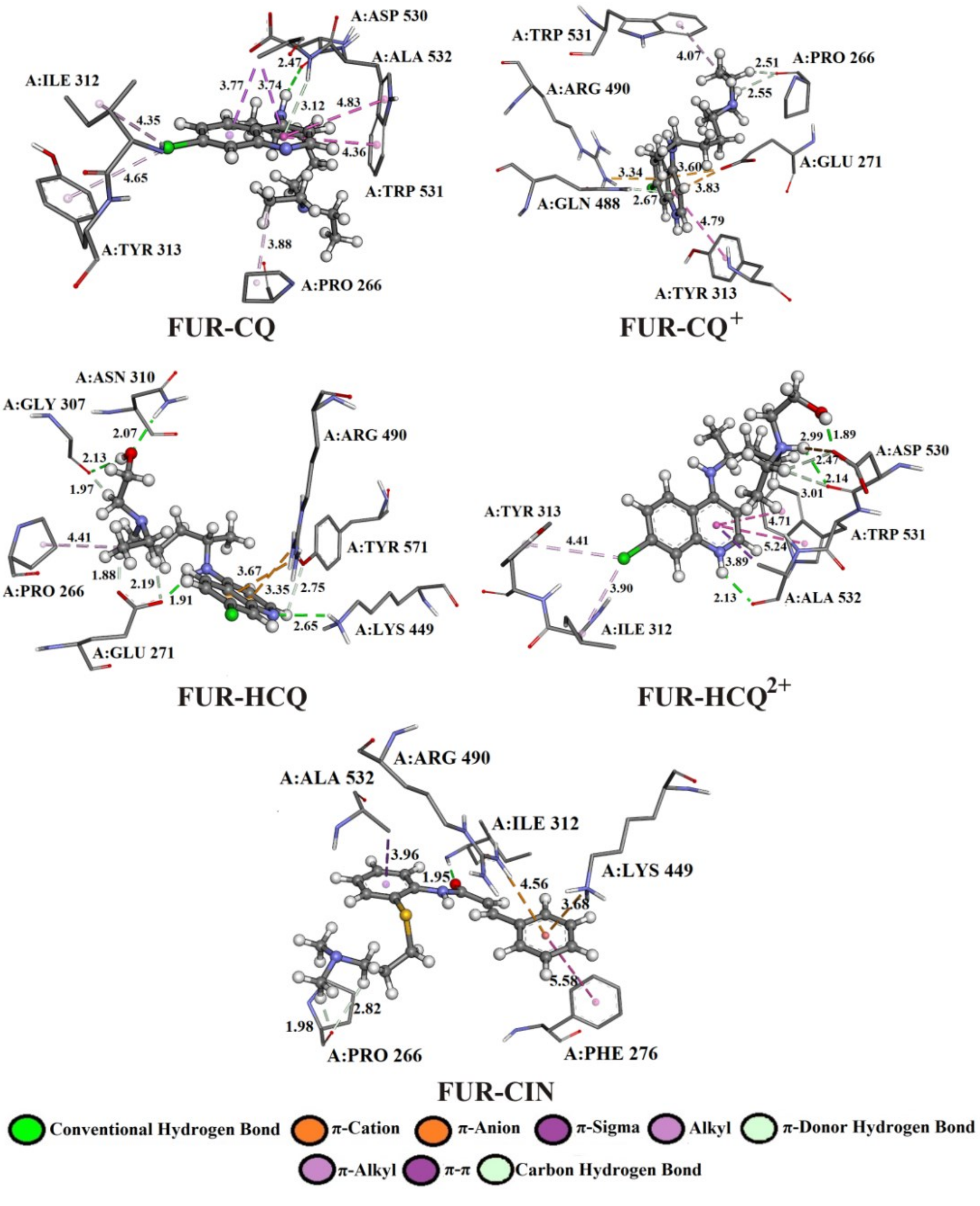

Fig. S9 The best docking positions of different acid-base forms of **CQ, HCQ, CIN** to **FUR** enzyme

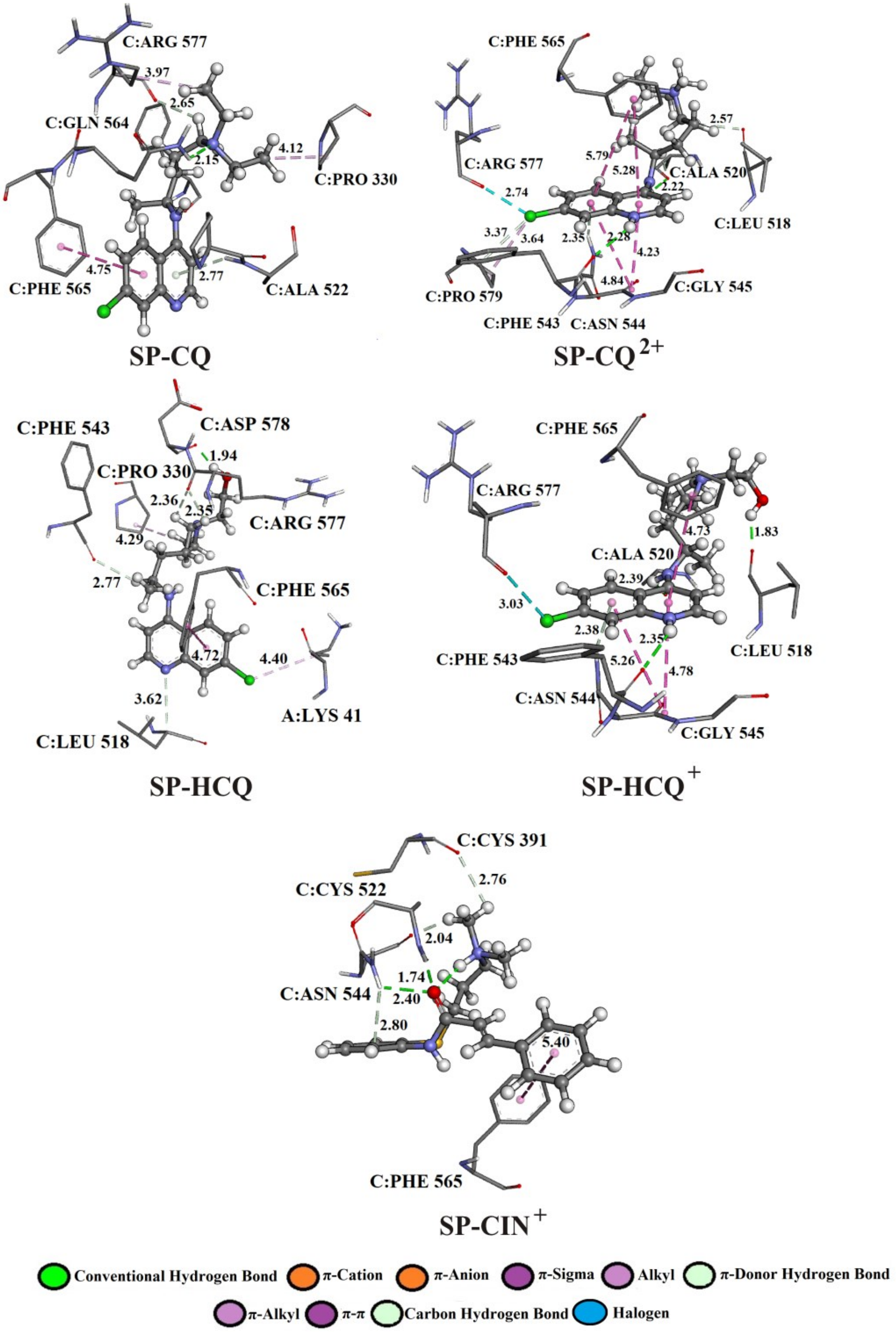

Fig. S10 The best docking positions of different acid-base forms of **CQ, HCQ, CIN** to **SP** enzyme

Table S2. Types of interactions and distance (Å) between amino acids of respective active sites of **FUR**, **SP** and acid-base forms of **Q** and **BZF**

**FUR-Q**			**SP-Q**		
Interactions:	Types of interactions:	**Å**	Interactions:	Types of interactions:	**Å**
A:ASN310:H – **Q**:O	Conventional Hydrogen Bond	**2.01**	**Q**:H - C:GLN1036:O	Conventional Hydrogen Bond	**2.93**
**Q**:H – A:TYR571:OH	Conventional Hydrogen Bond	**2.25**	**Q**:H - C:GLN1036:O	Conventional Hydrogen Bond	**2.25**
**Q**:H – A:TYR571:OH	Conventional Hydrogen Bond	**2.10**	**Q**:H - B:GLU1031:O	Conventional Hydrogen Bond	**1.94**
**Q**:H – A:GLY307:O	Conventional Hydrogen Bond	**2.33**	A:ARG1039:C - **Q**	π-σ	**3.29**
**Q**:H – A:ASP530:O	Conventional Hydrogen Bond	**2.19**	B:LYS1038:C,O;ARG 1039:N - :**Q**	Amide-π Stacked	**4.07**
A:ARG490:NH – **Q**	π-Cation	**3.28**	**Q** - A:LYS1038	π-Alkyl	**4.70**
A:GLU271:O – **Q**	π-Anion	**4.71**	**Q** - B:ARG1039	π-Alkyl	**4.45**
A:GLU271:O – **Q**	π-Anion	**4.02**			
A:ALA532:C – **Q**	π-σ	**3.88**	**Q** - B:LYS1038	π-Alkyl	**4.80**
A:ALA532:C – **Q**	π-σ	**3.45**			
A:TRP531 – **Q**	π-π-T-shaped	**5.26**	**Q** - B:LYS1038	π-Alkyl	**3.51**
A:TRP531 – **Q**	π-π-T-shaped	**4.79**			
**FUR-Q**$^{-}$			**SP-Q**$^{-}$		
A:ASN310:H - **Q**$^{-}$:O	Conventional Hydrogen Bond	**2.04**	B:LYS1038:H - **Q**$^{-}$:O	Conventional Hydrogen Bond	**2.42**
A:LYS449:H - **Q**$^{-}$:O	Conventional Hydrogen Bond	**2.61**			
**Q**$^{-}$:H - A:TYR571:OH	Conventional Hydrogen Bond	**2.13**	B:ARG1039:HN - **Q**$^{-}$:O	Conventional Hydrogen Bond	**2.77**
**Q**$^{-}$:H - A:GLY307:O	Conventional Hydrogen Bond	**2.31**			
**Q**$^{-}$:H - A:ASP530:O	Conventional Hydrogen Bond	**2.20**	**Q**$^{-}$:H - B:GLU1031:O	Conventional Hydrogen Bond	**2.24**
A:ARG490:NH - **Q**$^{-}$	π-Cation	**3.32**	B:GLY1035:C - **Q**$^{-}$:O	Carbon Hydrogen Bond	**3.51**
A:GLU271:O - **Q**$^{-}$	π-Anion	**4.62**			
A:GLU271:O - **Q**$^{-}$	π-Anion	**3.95**	A:LYS1038:N - **Q**$^{-}$	π-Cation	**3.68**
A:ALA532:C - **Q**$^{-}$	π-σ	**3.94**			
A:ALA532:C - **Q**$^{-}$	π-σ	**3.46**	A:ARG1039:C - **Q**$^{-}$	π-σ	**3.25**
A:TYR313 - **Q**$^{-}$	π-π Stacked	**5.47**			
A:TRP531 - **Q**$^{-}$	π-π Stacked	**5.31**	B:SER1037:C - **Q**$^{-}$	π-σ	**3.28**
A:TRP531 - **Q**$^{-}$	π-π Stacked	**4.83**			
**FUR-Q**$^{2-}$			**SP-Q**$^{2-}$		
A:ALA532:HN - **Q**$^{2-}$:O	Conventional Hydrogen Bond	**2.09**	B:GLN1036:HN - **Q**$^{2-}$:O	Conventional Hydrogen Bond	**2.29**
**Q**$^{2-}$:H - A:TYR313:OH	Conventional Hydrogen Bond	**1.80**	B:LYS1038:H - **Q**$^{2-}$:O	Conventional Hydrogen Bond	**2.30**
A:GLY307:CA - **Q**$^{2-}$:O	Carbon Hydrogen Bond	**3.05**	B:ARG1039:HN - **Q**$^{2-}$:O	Conventional Hydrogen Bond	**2.78**
A:ARG490:NH - **Q**$^{2-}$	π-Cation	**3.83**	**Q**$^{2-}$:H - B:GLU1031:O	Conventional Hydrogen	**2.22**

				Bond	
A:ARG490:NH - **Q**$^{2-}$	π-Cation	**3.02**	B:ARG1039:C - **Q**$^{2-}$:O	Carbon Hydrogen Bond	**3.77**
A:GLU271:O - **Q**$^{2-}$	π-Anion	**3.69**			
A:GLU271:O - **Q**$^{2-}$	π-Anion	**4.18**	A:LYS1038:N - **Q**$^{2-}$	π-Cation	**3.56**
A:GLN488:H - **Q**$^{2-}$	π-Donor Hydrogen Bond	**2.62**			
**Q**$^{2-}$:H - A:TRP531	π-Donor Hydrogen Bond	**2.96**	A:ARG1039:C - **Q**$^{2-}$	π-σ	**3.24**
A:ALA532:CB - **Q**$^{2-}$	π-σ	**3.92**			
A:TRP531 - **Q**$^{2-}$	π-π- T-shaped	**5.79**	B:SER1037:C - : **Q**$^{2-}$	π-σ	**3.25**
**FUR-BZF**			**SP-BZF**		
A:GLY265:HN - **BZF**:O	Conventional Hydrogen Bond	**2.90**	A:HIS1048:HN - **BZF**:O	Conventional Hydrogen Bond	**2.01**
A:ASN310:H - **BZF**:O	Conventional Hydrogen Bond	**1.84**	B:LYS1038:HN - **BZF**:O	Conventional Hydrogen Bond	**2.08**
A:ALA532:HN - **BZF**:O	Conventional Hydrogen Bond	**1.88**	**BZF**:H - A:SER1037:O	Conventional Hydrogen Bond	**2.07**
**BZF**:H - A:PRO266:O	Conventional Hydrogen Bond	**2.85**	**BZF**:H - A:SER1037:O	Conventional Hydrogen Bond	**2.12**
**BZF**:H - A:PRO266:O	Conventional Hydrogen Bond	**2.21**	**BZF**:H - B:GLU1031:O	Conventional Hydrogen Bond	**1.89**
**BZF**:H - A:GLY307:O	Conventional Hydrogen Bond	**2.34**	B:GLY1035:C - **BZF**:O	Carbon Hydrogen Bond	**3.20**
**BZF**:H - A:GLU271:O	Conventional Hydrogen Bond	**1.67**	A:LYS1038:O - **BZF**	π-Lone Pair	**2.83**
A:ILE312:HN - **BZF**	π-Donor Hydrogen Bond	**3.26**	B:GLN1036:O - **BZF**	π-Lone Pair	**2.92**
A:TRP531 - **BZF**	π-π Stacked	**4.71**	A:TYR1047 - **BZF**	π-π T-shaped	**5.36**
**FUR-BZF**$^{-}$			**SP-BZF**$^{-}$		
A:ASN310:H - **BZF**$^{-}$:O	Conventional Hydrogen Bond	**2.47**	C:GLN564:H - : **BZF**$^{-}$:O	Conventional Hydrogen Bond	**2.26**
A:ASN310:H - **BZF**$^{-}$:O	Conventional Hydrogen Bond	**2.49**	**BZF**$^{-}$:H - C:ARG577:O	Conventional Hydrogen Bond	**2.32**
A:ALA532:HN - **BZF**$^{-}$:O	Conventional Hydrogen Bond	**2.12**			
**BZF**$^{-}$:H - A:GLU271:O	Conventional Hydrogen Bond	**2.20**	**BZF**$^{-}$:H - C:ALA520:O	Conventional Hydrogen Bond	**2.37**
**BZF**$^{-}$:H - A:GLU271:O	Conventional Hydrogen Bond	**2.00**			
**BZF**$^{-}$:H - A:GLY307:O	Conventional Hydrogen Bond	**2.52**	C:PRO579:C - **BZF**$^{-}$:O	Carbon Hydrogen Bond	**2.85**
**BZF**$^{-}$:H - A:ASP530:O	Conventional Hydrogen Bond	**2.24**			
A:GLY307:C- **BZF**$^{-}$:O	Carbon Hydrogen Bond	**2.71**	C:PHE565 - **BZF**$^{-}$	π-π T-shaped	**5.17**
A:GLU271:O - **BZF**$^{-}$	π-Anion	**4.56**			
A:TRP531 - **BZF**$^{-}$	π-π Stacked	**5.77**	C:ASN544:C,O;GLY545:N - **BZF**$^{-}$	Amide-π Stacked	**4.38**
A:TRP531 - **BZF**$^{-}$	π-π Stacked	**4.51**			
**FUR-BZF**$^{2-}$			**SP-BZF**$^{2-}$		
A:ASN310:H - **BZF**$^{2-}$:O	Conventional Hydrogen Bond	**1.80**	A:HIS1048:HN-**BZF**$^{2-}$:O	Conventional Hydrogen Bond	**2.02**
A:ILE312:HN - **BZF**$^{2-}$:O	Conventional Hydrogen Bond	**2.00**	B:LYS1038:HN-**BZF**$^{2-}$:O	Conventional Hydrogen Bond	**2.16**

A:GLN488:H - **BZF²⁻**:O	Conventional Hydrogen Bond	**1.76**	**BZF²⁻**:H- A:SER1037:O	Conventional Hydrogen Bond	**2.15**
A:ALA532:HN - **BZF²⁻** :O	Conventional Hydrogen Bond	**2.01**	**BZF²⁻**:H- B:GLU1031:O	Conventional Hydrogen Bond	**1.85**
**BZF²⁻**:H - A:ASN310:O	Conventional Hydrogen Bond	**1.80**	B:GLY1035:C-**BZF²⁻** :O	Carbon Hydrogen Bond	**3.18**
**BZF²⁻**:H - A:ASP530:O	Conventional Hydrogen Bond	**2.54**	A:ARG1039:C-**BZF²⁻**	π-σ	**3.52**
A:GLY265:C - **BZF²⁻**:O	Carbon Hydrogen Bond	**3.73**	A:LYS1038:O-**BZF²⁻**	π-Lone Pair	**2.80**
A:GLY307:C- **BZF²⁻**:O	Carbon Hydrogen Bond	**2.85**	B:GLN1036:O-**BZF²⁻**	π-Lone Pair	**2.91**
			A:TYR1047-**BZF²⁻**	π-π- T-shaped	**5.35**

Table S3. Types of interactions and distance (Å) between amino acids of respective active sites of **FUR**, **SP** and acid-base forms of **CQ, HCQ,** and **CIN**

**FUR-CQ**			**SP-CQ**		
Interactions:	Types of interactions:	**Å**	Interactions:	Types of interactions:	**Å**
**CQ**:H - A:ASP530:O	Conventional Hydrogen Bond	**2.47**	C:GLN564:H - **CQ**:N	Conventional Hydrogen Bond	**2.15**
A:ALA532:HN - **CQ**	π-Donor Hydrogen Bond	**3.12**			
A:ALA532:C - **CQ**	π-σ	**3.74**	C:ALA522:HN - **CQ**	π-Donor Hydrogen Bond	**2.77**
A:ALA532:C - **CQ**	π-σ	**3.77**			
A:TRP531 - **CQ**	π-π- T-shaped	**4.83**	C:PHE565 - **CQ**	π-π- T-shaped	**4.75**
A:TRP531 - **CQ**	π-π- T-shaped	**4.36**			
**CQ**:Cl - A:ILE312	Alkyl	**4.35**	**CQ**:C - C:PRO330	Alkyl	**4.12**
**CQ**:C - A:PRO266	Alkyl	**3.88**			
A:TYR313 - **CQ**:Cl	π-Alkyl	**4.65**	**CQ**:C - C:ARG577	Alkyl	**3.97**
**FUR-CQ⁺**			**SP-CQ⁺**		
A:ARG490:NH - **CQ⁺**	π-Cation	**3.34**	**CQ⁺**:H - C:PHE543:O	Conventional Hydrogen Bond	**2.19**
A:GLU271:O - **CQ⁺**	π-Anion	**3.60**	**CQ⁺**:H - C:ALA520:O	Conventional Hydrogen Bond	**2.11**
A:GLU271:O - **CQ⁺**	π-Anion	**3.83**	C:PRO579:C - **CQ⁺**:CL	Carbon Hydrogen Bond	**3.73**
A:GLN488:H - **CQ⁺**	π-Donor Hydrogen Bond	**2.67**	C:ARG577:O - **CQ⁺**:CL	Halogen (Cl, Br, I)	**2.78**
A:TYR313 - **CQ⁺**	π-π Stacked	**4.79**	C:ASN544:H - **CQ⁺**	π-Donor Hydrogen Bond	**2.49**
**CQ⁺**:H - A:PRO266:O	Carbon Hydrogen Bond	**2.51**	C:PHE565 - **CQ⁺**	π-π- T-shaped	**5.56**
**CQ⁺**:H - A:PRO266:O	Carbon Hydrogen Bond	**2.55**	C:PHE565 - **CQ⁺**	π-π- T-shaped	**5.27**
A:TRP531 - **CQ⁺**:C	π-Alkyl	**4.07**	**CQ⁺**:CL - C:PRO579	Alkyl	**3.93**
**FUR-CQ²⁺**			**SP-CQ²⁺**		
**CQ²⁺**:N - A:GLU271:O	Attractive Charge	**4.47**	**CQ²⁺**:H - C:ALA520:O	Conventional Hydrogen Bond	**2.22**
**CQ²⁺**:H - A:GLU271:O	Conventional Hydrogen Bond	**2.12**	**CQ²⁺**:H - C:PHE543:O	Conventional Hydrogen Bond	**2.28**
**CQ²⁺**:H - A:PRO266:O	Conventional Hydrogen Bond	**1.95**	C:PRO579:C- **CQ²⁺**:CL	Carbon Hydrogen Bond	**3.37**

**CQ**$^{2+}$:H -A:GLU271:O	Carbon Hydrogen Bond	**2.48**	C:ARG577:O - **CQ**$^{2+}$:CL	Halogen (Cl, Br, I)	**2.74**
			C:ASN544:H - **CQ**$^{2+}$	π-Donor Hydrogen Bond	**2.35**
A:ARG490:NH - **CQ**$^{2+}$	π-Cation	**3.89**	C:PHE565 - **CQ**$^{2+}$	π-π- T-shaped	**5.79**
			C:PHE565 - **CQ**$^{2+}$	π-π- T-shaped	**5.28**
A:ARG490:NH - **CQ**$^{2+}$	π-Cation	**4.17**	C:ASN544:C,O;GLY 545:N - **CQ**$^{2+}$	Amide-π Stacked	**4.84**
			C:ASN544:C,O;GLY 545:N - **CQ**$^{2+}$	Amide-π Stacked	**4.23**
A:GLN488:H - **CQ**$^{2+}$	Pi-Donor Hydrogen Bond	**2.88**	**CQ**$^{2+}$:CL - C:PRO579	Alkyl	**3.64**
**FUR-HCQ**			**SP-HCQ**		
A:ASN310:H - **HCQ**:O	Conventional Hydrogen Bond	**2.07**	**HCQ**:H - C:ASP578:O	Conventional Hydrogen Bond	**1.94**
A:LYS449:H - **HCQ**:N	Conventional Hydrogen Bond	**2.65**	C:LEU518:C - **HCQ**:N	Carbon Hydrogen Bond	**3.62**
: **HCQ**:H - A:GLU271:O	Conventional Hydrogen Bond	**1.91**	**HCQ**:H - C:ARG577:O	Carbon Hydrogen Bond	**2.36**
**HCQ**:H - A:GLY307:O	Conventional Hydrogen Bond	**2.13**	**HCQ**:H - C:ARG577:O	Carbon Hydrogen Bond	**2.35**
**HCQ**:H - A:GLU271:O	Carbon Hydrogen Bond	**2.19**	**HCQ**:H - C:PHE543:O	Carbon Hydrogen Bond	**2.77**
**HCQ**:H - A:GLY307:O	Carbon Hydrogen Bond	**1.97**	C:PHE565 - **HCQ**	π-π- T-shaped	**4.72**
**HCQ**:H - A:TYR571:OH	Carbon Hydrogen Bond	**2.75**	**HCQ**:CL - A:LYS41	Alkyl	**4.40**
A:ARG490:NH - **HCQ**	π-Cation	**3.35**			
A:ARG490:NH - **HCQ**	π-Cation	**3.67**	**HCQ**:C - C:PRO330	Alkyl	**4.29**
**HCQ**:C - A:PRO266	Alkyl	**4.41**			
**FUR-HCQ**$^{+}$			**SP-HCQ**$^{+}$		
A:ALA532:HN - **HCQ**$^{+}$:O	Conventional Hydrogen Bond	**1.65**	**HCQ**$^{+}$:H - C:ALA520:O	Conventional Hydrogen Bond	**2.39**
**HCQ**$^{+}$:H - A:GLU271:O	Conventional Hydrogen Bond	**2.48**	**HCQ**$^{+}$:H - C:PHE543:O	Conventional Hydrogen Bond	**2.35**
**HCQ**$^{+}$:H - A:ASP530:O	Conventional Hydrogen Bond	**2.11**	**HCQ**$^{+}$:H - C:LEU518:O	Conventional Hydrogen Bond	**1.83**
**HCQ**$^{+}$:C - A:GLY307:O	Carbon Hydrogen Bond	**2.40**	C:ARG577:O - **HCQ**$^{+}$:CL	Halogen (Cl, Br, I)	**3.03**
A:ARG490:NH - **HCQ**$^{+}$	π-Cation	**3.81**	C:ASN544:H - **HCQ**$^{+}$	π-Donor Hydrogen Bond	**2.38**
A:ARG490:NH - **HCQ**$^{+}$	π-Cation	**4.10**	C:PHE565 - **HCQ**$^{+}$	π-π-T-shaped	**4.73**
A:GLN488:H - **HCQ**$^{+}$	π-Donor Hydrogen Bond	**2.72**	C:ASN544:C,O;GLY 545:N - **HCQ**$^{+}$	Amide-π Stacked	**5.26**
**HCQ**$^{+}$:C - A:PRO266	Alkyl	**3.94**	C:ASN544:C,O;GLY 545:N - **HCQ**$^{+}$	Amide-π Stacked	**4.78**
**FUR-HCQ**$^{2+}$			**SP-HCQ**$^{2+}$		
**HCQ**$^{2+}$:H - A:ASP530:O	Salt Bridge;Attractive Charge	**2.99**	C:GLN564:H - **HCQ**$^{2+}$:O	Conventional Hydrogen Bond	**2.07**
**HCQ**$^{2+}$:H - A:ALA532:O	Conventional Hydrogen Bond	**2.13**	**HCQ**$^{2+}$:H - C:ARG577:O	Conventional Hydrogen Bond	**2.02**

**HCQ**$^{2+}$:H - A:ASP530:O	Conventional Hydrogen Bond	**2.14**	**HCQ**$^{2+}$:C - C:ASN544:O	Carbon Hydrogen Bond	**2.06**
**HCQ**$^{2+}$:H - A:ASP530:O	Conventional Hydrogen Bond	**1.89**	C:PHE565 - **HCQ**$^{2+}$	π-π-T-shaped	**4.63**
A:ALA532:C - **HCQ**$^{2+}$	π-σ	**3.89**	**HCQ**$^{2+}$:CL - A:LYS41	Alkyl	**3.93**
A:TRP531 - **HCQ**$^{2+}$	π-π-T-shaped	**5.24**	**HCQ**$^{2+}$- C:LEU518	π-Alkyl	**4.32**
A:TRP531 - **HCQ**$^{2+}$	π-π-T-shaped	**4.71**	**HCQ**$^{2+}$:H - C:ARG577:O	Carbon Hydrogen Bond	**2.35**
**HCQ**$^{2+}$:Cl - A:ILE312	Alkyl	**3.89**	**HCQ**$^{2+}$:H - C:PHE543:O	Carbon Hydrogen Bond	**2.85**
A:TYR313 - **HCQ**$^{2+}$:Cl	π-Alkyl	**4.41**			
**FUR-CIN**			**SP-CIN**		
A:ILE312:HN - **CIN** :O	Conventional Hydrogen Bond	**1.95**	C:ALA522:HN - **CIN**:O	Conventional Hydrogen Bond	**1.85**
**CIN**:C - A:PRO266:O	Carbon Hydrogen Bond	**2.99**	C:ASN544:H - **CIN**:O	Conventional Hydrogen Bond	**2.34**
A:LYS449:N - **CIN**	π-Cation	**3.68**	**CIN**:H - C:ASN544:O	Carbon Hydrogen Bond	**2.25**
A:ARG490:NH - **CIN**	π-Cation	**4.56**	**CIN**:H - C:CYS391:O	Carbon Hydrogen Bond	**2.71**
A:ALA532:C - **CIN**	π-σ	**3.96**	C:ASN544:H - **CIN**	π-Donor Hydrogen Bond	**2.79**
A:PHE275 - **CIN**	π-π-T-shaped	**5.58**	C:PHE565 - **CIN**	π-π-T-shaped	**5.37**
			**CIN** - C:PRO579	π-Alkyl	**4.62**
**FUR-CIN**$^{+}$			**SP-CIN**$^{+}$		
**CIN**$^{+}$:N - A:ASP530:O	Attractive Charge	**5.14**	C:ALA522:HN - **CIN**$^{+}$:O	Conventional Hydrogen Bond	**1.74**
A:GLN488:H - **CIN**$^{+}$:O	Conventional Hydrogen Bond	**1.85**	C:ASN544:H - :UNL1:O	Conventional Hydrogen Bond	**2.40**
A:ARG490:H - **CIN**$^{+}$:O	Conventional Hydrogen Bond	**2.80**	**CIN**$^{+}$:H21 - C:ASN544:O	Carbon Hydrogen Bond	**2.04**
A:TYR571:H - **CIN**$^{+}$:O	Conventional Hydrogen Bond	**2.55**	**CIN**$^{+}$:H22 - C:CYS391:O	Carbon Hydrogen Bond	**2.76**
**CIN**$^{+}$:H - A:GLU271:O	Conventional Hydrogen Bond	**1.78**	C:ASN544:H - **CIN**$^{+}$	π-Donor Hydrogen Bond	**2.80**
**CIN**$^{+}$:H - A:ASP530:O	Conventional Hydrogen Bond	**1.93**	C:PHE565 - **CIN**$^{+}$	π-π-T-shaped	**5.40**
**CIN**$^{+}$:H - A:ASN529:O	Carbon Hydrogen Bond	**2.93**	C:PHE565 - **CIN**$^{+}$	π-π-T-shaped	**5.40**
**CIN**$^{+}$:H - A:ASP530:O	Carbon Hydrogen Bond	**2.60**			
A:LYS449:H - **CIN**$^{+}$	π-Cation	**2.55**	C:PHE565 - **CIN**$^{+}$	π-π-T-shaped	**5.40**
**CIN**$^{+}$:N - A:TRP531	π-Cation	**4.87**			

Cartesian coordinates

Q

0 1

C	2.19420800	-1.43626600	-0.51847200
C	1.83418800	-0.16655900	-0.05577900
C	2.83142300	0.71781300	0.37665300
C	4.15989300	0.33761100	0.33396400
C	4.51560000	-0.92978800	-0.14199100
C	3.53038900	-1.81150700	-0.56328300
C	0.41808100	0.21549700	-0.02784400
C	-0.09307800	1.46912500	-0.12619100
C	-1.52338700	1.69670300	-0.12685700
C	-2.35437900	0.52867100	-0.02381500
C	-1.75668000	-0.73531900	0.07389500
O	-0.40792500	-0.86237800	0.07617300
C	-3.77009300	0.59911700	-0.01942400
C	-4.52716000	-0.55000900	0.08234700
C	-3.88033200	-1.78866800	0.18161800
C	-2.49610100	-1.90278700	0.17917700
O	-4.39809000	1.79086500	-0.11334100
O	-4.59124500	-2.93870800	0.28432400
O	-1.96050200	2.86178700	-0.23141800
O	0.69671300	2.56786300	-0.26205500
O	5.84925400	-1.21355200	-0.14838400
H	2.58938700	1.69721300	0.76612800
H	1.43497500	-2.12956800	-0.85589600
H	0.09884700	3.32688300	-0.36540400
H	-2.01003000	-2.86597600	0.25787400
H	-5.60936100	-0.49087100	0.08417200
H	6.00145900	-2.10720800	-0.48200100
H	-3.71690200	2.49180300	-0.17931100
H	-5.53794100	-2.74596700	0.28314900
H	3.82056100	-2.79074800	-0.92830100
O	5.11740700	1.21301400	0.76839500
H	5.98867400	0.80150400	0.68604700

Q^-

-1 1

C	2.22420400	-1.47329600	-0.47382100
C	1.86330900	-0.18311500	-0.05772000
C	2.88759600	0.70873200	0.33604500

C	4.19700500	0.30361700	0.29704600
C	4.59997300	-1.00092600	-0.13527000
C	3.55218600	-1.86973100	-0.51850500
C	0.46215400	0.20403300	-0.03250700
C	-0.05611200	1.46495300	-0.11445500
C	-1.47367000	1.69820800	-0.10806800
C	-2.31594800	0.53403800	-0.01589500
C	-1.72653100	-0.73309100	0.05950200
O	-0.37841800	-0.86694800	0.05870100
C	-3.72927200	0.61410000	-0.00615200
C	-4.49522700	-0.53129500	0.08301800
C	-3.85674000	-1.77519600	0.16106700
C	-2.47352300	-1.89819100	0.14841400
O	-4.34719400	1.81360400	-0.08389000
O	-4.57605200	-2.92303500	0.25458900
O	-1.91617000	2.86870300	-0.20295700
O	0.74270200	2.56506900	-0.25078100
O	5.85694800	-1.29572100	-0.14552800
H	2.65882400	1.70480800	0.69087600
H	1.45430000	-2.17057500	-0.78176000
H	0.14462200	3.32411200	-0.34802700
H	-1.99327600	-2.86550200	0.21139900
H	-5.57693900	-0.46393200	0.09062400
H	-3.65355700	2.50501700	-0.14868800
H	-5.52080800	-2.72185500	0.26553200
H	3.81250000	-2.86782100	-0.85474100
O	5.20495200	1.15523000	0.69004200
H	6.02377700	0.64349400	0.57717500

Q^{2-}

-2 1

C	2.21822400	-1.46022800	-0.50096600
C	1.83748900	-0.18545800	-0.06195300
C	2.84649900	0.71108700	0.35450200
C	4.16307900	0.32522300	0.31300400
C	4.58648000	-0.96301800	-0.14233200
C	3.55400000	-1.83779000	-0.54609700
C	0.42699400	0.18606300	-0.03599500
C	-0.10241800	1.43546300	-0.12329400
C	-1.53098200	1.65386900	-0.11435900
C	-2.35339000	0.49675000	-0.01622000
C	-1.76050400	-0.77934600	0.06605900
O	-0.40111000	-0.89674400	0.06348800

C	-3.77648200	0.55096500	-0.00290500
C	-4.52947400	-0.58916200	0.09369000
C	-3.91703500	-1.88619200	0.18367200
C	-2.49180200	-1.94134000	0.16129700
O	-4.39748400	1.76120500	-0.08796200
O	-4.62793900	-2.94623200	0.28117600
O	-1.96890300	2.83384600	-0.21298300
O	0.67884100	2.55054000	-0.26779100
O	5.85166200	-1.23889700	-0.15234800
H	2.60104700	1.69694500	0.72733700
H	1.45947500	-2.16234500	-0.82572000
H	0.06054400	3.29392700	-0.36443100
H	-1.98652400	-2.89708500	0.22791100
H	-5.61127000	-0.51832600	0.10324200
H	-3.69978500	2.44704900	-0.15638700
H	3.82932500	-2.82613600	-0.89920900
O	5.15747100	1.18391000	0.72753800
H	5.98280400	0.68363100	0.60969800

BZF

0 1

C	-1.77310300	0.29215800	-0.85374100
C	-1.84577400	0.36857800	0.54095100
C	-2.52532400	-0.58325900	-1.59683900
C	-2.72287300	-0.47117400	1.24479800
C	-3.39760300	-1.40611900	-0.86429100
C	-3.50703700	-1.36056100	0.53195000
O	-0.83760600	1.14409400	-1.37980700
C	-0.31056300	1.95487200	-0.32004300
C	-0.88910800	1.33763900	0.98357300
C	1.24026000	1.96758500	-0.42739700
C	2.03612600	0.74223200	-0.25446300
C	3.66578900	-1.49298200	0.08484700
C	4.23787000	-0.23317600	-0.05187500
C	2.27130700	-1.63782100	0.05220700
C	3.42899900	0.87659900	-0.22390400
C	1.46274200	-0.53054200	-0.11233700
O	1.74197300	3.05071300	-0.65098300
O	-0.57390700	1.69812200	2.10070000
O	4.46589900	-2.57323200	0.24657300
O	-0.79820300	3.24363500	-0.40883000
O	-4.18925200	-2.30447000	-1.48802600

O	-2.81406500	-0.43303300	2.58829800
O	1.80763400	-2.91533600	0.18773000
H	-2.45782700	-0.64735400	-2.67549700
H	-4.19626900	-2.02248000	1.04025600
H	5.31699000	-0.14380200	-0.02120200
H	3.87282500	1.85828300	-0.32906900
H	0.39427700	-0.70243800	-0.14088400
H	3.92374600	-3.37150100	0.32654700
H	-0.09610100	3.79895000	-0.78927400
H	-4.05108600	-2.28240800	-2.44487700
H	-2.20070700	0.22380800	2.95133400
H	0.84413400	-2.93917300	0.11682300

BZF⁻

-1 1

C	-1.79734400	0.24003600	-0.88851000
C	-1.86152100	0.34380800	0.51707800
C	-2.50918900	-0.65962500	-1.61585600
C	-2.71901500	-0.52995000	1.22816800
C	-3.39637100	-1.55095100	-0.89837900
C	-3.46647400	-1.44772700	0.54243100
O	-0.87922800	1.12574100	-1.41597200
C	-0.37636400	1.94390300	-0.35585100
C	-0.94862000	1.32249200	0.94720400
C	1.17259300	1.97797800	-0.43746500
C	1.98148000	0.75964800	-0.25355000
C	3.63238300	-1.45396800	0.12477900
C	4.18342300	-0.17853000	0.08594000
C	2.24896900	-1.62519800	-0.02632200
C	3.36392500	0.92066400	-0.10636100
C	1.42901400	-0.52944900	-0.21165400
O	1.67387800	3.06773400	-0.63452400
O	-0.63470500	1.71891300	2.07000600
O	4.44158600	-2.52503200	0.30994000
O	-0.87681700	3.23227100	-0.45767100
O	-4.09519700	-2.40526800	-1.51872500
O	-2.79311900	-0.45153100	2.58341600
O	1.80706500	-2.91735600	0.01981300
H	-2.43390300	-0.72638500	-2.69367600
H	-4.12963500	-2.11945400	1.07465700
H	5.25392100	-0.06771800	0.21037900
H	3.79038500	1.91518300	-0.13325200
H	0.37050300	-0.71840700	-0.33605900

H	3.91120400	-3.33514100	0.31342700
H	-0.17374000	3.79450300	-0.82351100
H	-2.20360900	0.24893100	2.90169200
H	0.84583900	-2.95406500	-0.07382500

BZF^{2-}

-2 1

C	-1.82450500	0.31469000	-0.84505500
C	-1.79465400	0.34708300	0.56622700
C	-2.58308100	-0.54016900	-1.57453300
C	-2.61413300	-0.56262900	1.28198000
C	-3.43207500	-1.46532900	-0.85069100
C	-3.40835600	-1.43840900	0.59531900
O	-0.93246000	1.22280600	-1.38695000
C	-0.34797200	1.97239700	-0.32229600
C	-0.86217800	1.30880500	0.98739400
C	1.18237800	1.91344000	-0.50895900
C	1.95060800	0.62963900	-0.29193600
C	3.68034200	-1.57038500	0.16016500
C	4.19608600	-0.20799300	0.27978900
C	2.19034000	-1.71803200	-0.20066200
C	3.36865500	0.81893300	0.05879200
C	1.36816900	-0.58641100	-0.44122500
O	1.77823300	2.90961400	-0.81931500
O	-0.47872900	1.66355600	2.10150700
O	4.29592000	-2.59242100	0.31241100
O	-0.77419200	3.28445500	-0.32643300
O	-4.17232000	-2.28124600	-1.46973000
O	-2.60469100	-0.56575000	2.64032000
O	1.72569300	-2.88531700	-0.27160000
H	-2.57337900	-0.54854000	-2.65670800
H	-4.03929900	-2.13698300	1.13194800
H	5.23930200	-0.08507500	0.54124000
H	3.71856100	1.84039000	0.13596500
H	0.33967200	-0.75768900	-0.72863200
H	-0.21830600	3.80126400	-0.93022600
H	-1.99567300	0.11225800	2.96860100
H	2.41173900	-3.56497600	-0.06973500

CQ

0 1

C	2.82501400	0.16372000	0.24687300

C	1.45938900	0.25185600	0.71153700
C	3.56475100	-1.00576800	0.56253000
N	0.71990300	1.35747800	0.47893600
C	4.90814800	-1.10965700	0.11949700
C	3.44932300	1.18607800	-0.50685800
N	3.05979100	-2.05657500	1.27438200
C	5.46623000	-0.09327400	-0.60319100
C	0.97020700	-0.84393300	1.42018100
C	4.74886100	1.07180000	-0.93163900
C	1.80555000	-1.93787000	1.66579100
N	-5.19534000	-0.56346400	-0.30570300
Cl	7.12471200	-0.22979700	-1.14678900
C	-0.70254200	1.47949800	0.79899400
C	-3.00229100	0.47965600	0.29745700
C	-3.77045800	-0.44653700	-0.63815700
C	-5.75073000	-1.73692700	-0.99323700
C	-5.92095400	0.66331900	-0.69427800
C	-1.55886200	0.65425200	-0.16889500
C	-1.06815200	2.95667700	0.77403600
C	-7.21179900	-2.00932000	-0.67217900
C	-6.90810300	1.14652600	0.35961700
H	1.11058300	2.09543600	-0.08753900
H	5.46899800	-2.00281400	0.36587000
H	2.91167100	2.08796000	-0.77395100
H	-0.04443700	-0.87125200	1.79203200
H	5.21823300	1.85834100	-1.50834100
H	1.39887600	-2.77428800	2.22782500
H	-0.84757800	1.10047500	1.81582100
H	-3.49994300	1.45190300	0.35946300
H	-3.00920500	0.05218700	1.30662600
H	-3.64429600	-0.10722600	-1.68120200
H	-3.32627800	-1.44401000	-0.57611100
H	-5.15514300	-2.60457600	-0.69917500
H	-5.63626900	-1.62276500	-2.08526900
H	-6.43751200	0.50059900	-1.65064900
H	-5.19641700	1.45955900	-0.87740500
H	-1.10504900	-0.33503900	-0.29235800
H	-1.53533200	1.14093200	-1.15115700
H	-0.46948900	3.51879400	1.49344200
H	-2.12064300	3.09489300	1.02284400
H	-0.89531200	3.37026300	-0.22443800
H	-7.36384600	-2.12134600	0.40436000
H	-7.52124800	-2.93734900	-1.15845900
H	-7.86641700	-1.21327200	-1.03252400

H	-7.65323400	0.38441600	0.59442400
H	-7.43150500	2.03942500	0.00703100
H	-6.38127400	1.40264400	1.28248300

CQ^+

1 1

C	2.81888600	0.16858600	0.19129600
C	1.42511800	0.23207100	0.59803800
C	3.56643000	-0.97981900	0.51088900
N	0.69903000	1.31600100	0.36191400
C	4.92009700	-1.09502200	0.15154100
C	3.46775800	1.20269600	-0.51714400
N	2.96537100	-2.01547800	1.18269600
C	5.50204900	-0.05830400	-0.53002500
C	0.88152100	-0.89435300	1.25850600
C	4.78864300	1.10038500	-0.87703800
C	1.67465900	-1.97518700	1.53323000
N	-5.22949600	-0.54033700	-0.35119900
Cl	7.17757500	-0.17532400	-0.98955700
C	-0.72527500	1.46701600	0.69274300
C	-3.01513200	0.46668600	0.21355300
C	-3.85103100	-0.31041500	-0.79615500
C	-5.84191200	-1.58171700	-1.18576200
C	-6.01669500	0.70884500	-0.40970900
C	-1.59671200	0.69367700	-0.30351900
C	-1.04587300	2.95364900	0.71540400
C	-7.24341300	-1.97336700	-0.74453700
C	-6.84466700	0.96235200	0.84236200
H	1.13051100	2.10246700	-0.10612400
H	5.47207100	-1.99007300	0.41398100
H	2.93374300	2.10015100	-0.80357900
H	-0.15226100	-0.92560300	1.56788800
H	5.27739200	1.89777800	-1.42144700
H	1.29613600	-2.84799300	2.04866200
H	-0.87187500	1.05831900	1.69671500
H	-3.49089700	1.42519800	0.44061600
H	-2.97002600	-0.09939900	1.15066200
H	-3.83791500	0.21301500	-1.76807100
H	-3.38210600	-1.28555500	-0.95589200
H	-5.19629700	-2.46232300	-1.14333900
H	-5.87122800	-1.25424800	-2.23948600
H	-6.66715400	0.69545600	-1.29466800
H	-5.33654600	1.55160100	-0.55153600

H	-1.13619800	-0.27799900	-0.51106100
H	-1.61728700	1.24677200	-1.24942600
H	-0.44278300	3.47262700	1.46266600
H	-2.09780700	3.10625900	0.95753000
H	-0.85208700	3.39650300	-0.26598400
H	-7.24776000	-2.30296300	0.29756700
H	-7.60181300	-2.79822800	-1.36471800
H	-7.95175300	-1.14872100	-0.84810400
H	-7.54248900	0.14515200	1.03500900
H	-7.41974200	1.88628600	0.73676300
H	-6.19263600	1.06432300	1.71387900
H	3.51897200	-2.83626900	1.40805400

CQ^{2+}

2 1

C	2.83316100	0.16335000	0.19917400
C	1.44072400	0.21940000	0.61105500
C	3.58695100	-0.98281800	0.51229900
N	0.70930500	1.30116700	0.37692400
C	4.93928300	-1.09213100	0.14618300
C	3.47516100	1.20203400	-0.50908100
N	2.99317900	-2.02245400	1.18437500
C	5.51430200	-0.05123400	-0.53483400
C	0.90552300	-0.90962800	1.27265400
C	4.79477800	1.10585800	-0.87484900
C	1.70459800	-1.98794200	1.54167100
N	-5.21081000	-0.52576000	-0.43298200
Cl	7.18776400	-0.16050000	-1.00266600
C	-0.71546700	1.44934200	0.70328300
C	-3.01826100	0.52021400	0.15139000
C	-3.78633800	-0.31872500	-0.85514100
C	-5.85850100	-1.62790000	-1.22291200
C	-6.01464100	0.75883100	-0.47940000
C	-1.57231900	0.68485000	-0.31190300
C	-1.03556400	2.93583700	0.74093300
C	-7.20476600	-2.01453300	-0.64576700
C	-6.56442500	1.10895400	0.88709100
H	1.13708200	2.08864400	-0.09289400
H	5.49571700	-1.98593300	0.40347900
H	2.93702600	2.09839500	-0.79097200
H	-0.12605100	-0.94608300	1.58836900
H	5.27804800	1.90696200	-1.41870300
H	1.33230000	-2.86298100	2.05787600

H	-0.87056200	1.02819100	1.70063800
H	-3.49018900	1.49766100	0.27799700
H	-3.03584600	0.01558100	1.12430000
H	-3.81966300	0.14802400	-1.84155700
H	-3.33705300	-1.30820700	-0.94648800
H	-5.16951700	-2.47140300	-1.19597400
H	-5.93369100	-1.26379800	-2.24843200
H	-6.80855400	0.61360500	-1.20988600
H	-5.35587900	1.53899700	-0.85583900
H	-1.13868200	-0.30640000	-0.47802700
H	-1.53976100	1.21722600	-1.26861200
H	-0.41750900	3.44640200	1.48160100
H	-2.08172600	3.09122400	1.00476200
H	-0.85740500	3.38603600	-0.24001200
H	-7.10308700	-2.32382600	0.39713500
H	-7.59349800	-2.85983200	-1.21527700
H	-7.93209000	-1.20363300	-0.70652100
H	-7.22798600	0.32330100	1.25510000
H	-7.13204700	2.03781400	0.81875400
H	-5.75333500	1.25080700	1.60531800
H	3.55105100	-2.84166900	1.40529800
H	-5.18599000	-0.83984000	0.54459200

HCQ

0 1

C	3.11371000	0.17868100	0.23924200
C	1.72867500	0.27953300	0.63811600
C	3.84911000	-0.96180200	0.65506900
N	0.98377300	1.34867000	0.27658400
C	5.20629100	-1.08500100	0.26194700
C	3.76449500	1.16816900	-0.53546500
N	3.32823500	-1.96252300	1.42516500
C	5.78549000	-0.10707400	-0.49650400
C	1.23109600	-0.75382100	1.42868600
C	5.07922900	1.03927600	-0.90524600
C	2.06614400	-1.81891800	1.78108700
N	-4.88638500	-0.73223200	-0.49129500
Cl	7.46136200	-0.26655500	-0.97728300
C	-0.45089100	1.47800700	0.53462700
C	-2.74238300	0.47867200	0.00554500
C	-3.46487900	-0.57611500	-0.82265800
C	-5.43729200	-1.86526800	-1.24829200
C	-5.62716200	0.50696600	-0.78087100

C	-1.26434900	0.54909400	-0.37399800
C	-0.83127000	2.94097000	0.35501000
C	-6.80387400	-2.31334700	-0.75400900
C	-5.99536900	1.30658300	0.45186600
H	1.35687200	1.98096600	-0.41699000
H	5.76195900	-1.96007700	0.57600400
H	3.23827600	2.06196500	-0.84840100
H	0.20587300	-0.76083400	1.77176200
H	5.57038000	1.80224900	-1.49529200
H	1.64987700	-2.61145000	2.39696400
H	-0.62359000	1.19777800	1.57850600
H	-3.20813300	1.45670600	-0.14272800
H	-2.83678900	0.23272100	1.06993400
H	-3.34894900	-0.33163000	-1.89394700
H	-2.97732900	-1.54140900	-0.66030700
H	-4.73661300	-2.69727000	-1.14660000
H	-5.48825700	-1.62201300	-2.32286200
H	-6.55604600	0.26963600	-1.30559300
H	-5.04918500	1.15125200	-1.45646000
H	-0.83466400	-0.45712100	-0.32874500
H	-1.15716100	0.89772100	-1.40794500
H	-0.23681500	3.57894300	1.01189900
H	-1.88455500	3.09665600	0.58926900
H	-0.66049300	3.25033000	-0.68102500
H	-6.75946800	-2.59389200	0.30111900
H	-7.13300900	-3.18444400	-1.32566300
H	-7.56176200	-1.53599800	-0.86931900
H	-6.36846200	2.28939800	0.14333800
H	-5.12403800	1.45066900	1.09678300
O	-7.01532900	0.60057400	1.16357900
H	-7.17842600	1.06096500	1.99339000

HCQ+

1 1

C	3.11567700	0.16588700	0.20177400
C	1.72594700	0.20969400	0.62687800
C	3.91828400	-0.91295400	0.61661700
N	0.95055200	1.23394900	0.29922000
C	5.26975200	-1.00578100	0.24292200
C	3.70776200	1.15128800	-0.61708000
N	3.37477400	-1.90184800	1.39884100
C	5.79553600	-0.01689700	-0.54712800
C	1.24454100	-0.86783900	1.40629600

C	5.02623600	1.07046600	-0.99098500
C	2.08906900	-1.88123800	1.76987000
N	-4.95499800	-0.67889300	-0.54864800
Cl	7.46727900	-0.10674700	-1.02510600
C	-0.47898900	1.36176600	0.61933800
C	-2.78711600	0.44250900	0.03674500
C	-3.53071300	-0.53141000	-0.86876100
C	-5.53747800	-1.72906300	-1.39549900
C	-5.66641700	0.59787300	-0.72934500
C	-1.30780000	0.49993700	-0.33763400
C	-0.84331400	2.83739700	0.55389600
C	-6.90524400	-2.19155900	-0.91814700
C	-6.01896100	1.29207500	0.57002500
H	1.34238400	1.98486400	-0.25408100
H	5.86392700	-1.84790100	0.57821200
H	3.13264700	1.99417500	-0.97944000
H	0.21816500	-0.91257200	1.73679300
H	5.47010800	1.83005400	-1.62106900
H	1.75781900	-2.71433700	2.37555800
H	-0.61973700	1.00896900	1.64447300
H	-3.22634000	1.44010600	-0.04546700
H	-2.89186100	0.12475200	1.08060400
H	-3.40651300	-0.20863900	-1.91818200
H	-3.06553900	-1.51708200	-0.77864200
H	-4.85326300	-2.58060200	-1.38098600
H	-5.59923500	-1.38789200	-2.44233500
H	-6.59901900	0.42751500	-1.27341200
H	-5.07251200	1.28495500	-1.34613800
H	-0.89911700	-0.51616200	-0.34517200
H	-1.19104200	0.90137000	-1.35076600
H	-0.20849900	3.42302800	1.22153300
H	-1.88014200	2.98453600	0.85586400
H	-0.72348300	3.21306600	-0.46660800
H	-6.84327100	-2.59045900	0.09717400
H	-7.27815800	-2.98122900	-1.57471300
H	-7.63821600	-1.38225000	-0.92178800
H	-6.36730600	2.30783000	0.35326200
H	-5.14582300	1.35391400	1.22570000
O	-7.05786300	0.54832500	1.21264800
H	-7.21613500	0.93621100	2.07974100
H	3.96641500	-2.67345500	1.69091800

HCQ^{2+}

2 1

C	3.11198000	0.16860400	0.19531200
C	1.71488500	0.24658700	0.58733900
C	3.86792100	-0.94708900	0.60014500
N	0.98034000	1.30123800	0.25917900
C	5.22472300	-1.07450300	0.25820200
C	3.75593300	1.15592800	-0.58111400
N	3.27209200	-1.93789600	1.34085000
C	5.80210900	-0.08268500	-0.49085100
C	1.18025800	-0.82918000	1.33270900
C	5.08040700	1.04138100	-0.92375100
C	1.98129000	-1.88070200	1.68787100
N	-4.92780000	-0.57201400	-0.65267700
Cl	7.48217500	-0.21444800	-0.92884200
C	-0.45172400	1.46795200	0.54318100
C	-2.75644100	0.56615400	-0.05179200
C	-3.49041500	-0.34106000	-1.02312300
C	-5.55493600	-1.56447200	-1.59643100
C	-5.72020600	0.70785300	-0.57145100
C	-1.27894000	0.62704800	-0.43490600
C	-0.77195100	2.95309400	0.46259000
C	-6.82150500	-2.16609300	-1.02404100
C	-5.91998500	1.14092800	0.86243600
H	1.41213100	2.05287300	-0.26248800
H	5.78242400	-1.94414000	0.58601300
H	3.21734900	2.02691100	-0.93348400
H	0.14722700	-0.84321700	1.64541600
H	5.56599100	1.80272600	-1.52000700
H	1.60910600	-2.71524300	2.26732800
H	-0.62947200	1.12024500	1.56449700
H	-3.18833100	1.56893600	-0.06857100
H	-2.85800400	0.17221200	0.96590200
H	-3.50110600	0.07441700	-2.03305300
H	-3.02323700	-1.32628300	-1.05007800
H	-4.80802200	-2.33831500	-1.76808500
H	-5.72904100	-1.02873200	-2.53068100
H	-6.68901100	0.53078400	-1.03589000
H	-5.19403500	1.46802900	-1.14763900
H	-0.87687100	-0.39046200	-0.46488000
H	-1.16676700	1.05069300	-1.43898800
H	-0.15058500	3.52006600	1.15814900
H	-1.81640000	3.13239300	0.71702400
H	-0.59485000	3.32294500	-0.55155600
H	-6.61598900	-2.66058200	-0.07197700

H	-7.19157400	-2.91574800	-1.72497800
H	-7.60915600	-1.42660800	-0.87624500
H	-6.49320200	2.07199100	0.85708600
H	-4.96609000	1.31856900	1.36433000
O	-6.63627900	0.09943000	1.51795300
H	-6.61908200	0.26507900	2.46675200
H	3.83129700	-2.73633800	1.62526600
H	-4.94428600	-1.00079200	0.28210400
